# Progressive nitrogen limitation across the Tibetan alpine permafrost region

**DOI:** 10.1038/s41467-020-17169-6

**Published:** 2020-07-03

**Authors:** Dan Kou, Guibiao Yang, Fei Li, Xuehui Feng, Dianye Zhang, Chao Mao, Qiwen Zhang, Yunfeng Peng, Chengjun Ji, Qiuan Zhu, Yunting Fang, Xueyan Liu, Xu-Ri, Siqi Li, Jia Deng, Xunhua Zheng, Jingyun Fang, Yuanhe Yang

**Affiliations:** 1 State Key Laboratory of Vegetation and Environmental Change, Institute of Botany, Chinese Academy of Sciences, Beijing, 100093 China; 2 University of Chinese Academy of Sciences, Beijing, 100049 China; 3 Biogeochemistry Research Group, Department of Biological and Environmental Sciences, University of Eastern Finland, Kuopio, 70210 Finland; 4 Earth, Atmospheric and Planetary Sciences, Purdue University, West Lafayette, IN 47907 USA; 5 Institute of Ecology, College of Urban and Environmental Sciences, and Key Laboratory for Earth Surface Processes of the Ministry of Education, Peking University, Beijing, 100871 China; 6 College of Hydrology and Water Resources, Hohai University, Nanjing, 210098 China; 7 CAS Key Laboratory of Forest Ecology and Management, Institute of Applied Ecology, Chinese Academy of Sciences, Shenyang, 110016 China; 8 School of Earth System Science, Tianjin University, Tianjin, 300072 China; 9 Key Laboratory of Alpine Ecology and Biodiversity, Institute of Tibetan Plateau Research, Chinese Academy of Sciences, Beijing, 100101 China; 10 State Key Laboratory of Atmospheric Boundary Layer Physics and Atmospheric Chemistry, Institute of Atmospheric Physics, Chinese Academy of Sciences, Beijing, 100029 China; 11 Earth Systems Research Centre, Institute for the Study of Earth, Oceans and Space, University of New Hampshire, Durham, NH 03824 USA

**Keywords:** Element cycles, Element cycles

## Abstract

The ecosystem carbon (C) balance in permafrost regions, which has a global significance in understanding the terrestrial C-climate feedback, is significantly regulated by nitrogen (N) dynamics. However, our knowledge on temporal changes in vegetation N limitation (i.e., the supply of N relative to plant N demand) in permafrost ecosystems is still limited. Based on the combination of isotopic observations derived from a re-sampling campaign along a ~3000 km transect and simulations obtained from a process-based biogeochemical model, here we detect changes in ecosystem N cycle across the Tibetan alpine permafrost region over the past decade. We find that vegetation N limitation becomes stronger despite the increased available N production. The enhanced N limitation on vegetation growth is driven by the joint effects of elevated plant N demand and gaseous N loss. These findings suggest that N would constrain the future trajectory of ecosystem C cycle in this alpine permafrost region.

## Abstract (web summary)

Massive stores of carbon and nutrients in permafrost could be released by global warming. Here the authors show that though warming across the Tibetan alpine permafrost region accelerates nitrogen liberation, contrary to expectations the elevated nutrients do not alleviate plant nitrogen limitation.

## Introduction

Permafrost regions store more than 1300 Pg (1 Pg = 10^15^ g) carbon (C, ca. 43% of the global soil organic C storage) in ~15% of the global land area^1,2^. During the past decades, permafrost regions have experienced two times faster climate warming compared with the rest of Earth’s areas^3,4^, which has triggered extensive permafrost thaw^1,4^. Consequently, large amounts of C stored in permafrost regions may be emitted into the atmosphere as carbon dioxide (CO_2_) and methane (CH_4_) because of the accelerated microbial decomposition^5–7^, potentially aggravating global warming through the positive C-climate feedback^4^. However, permafrost regions may still appear as a C sink due to the enhanced vegetation productivity under both the current and near-future climate scenarios^8,9^. These uncertainties in ecosystem C balance across permafrost regions could be partly ascribed to ecosystem nitrogen (N) dynamics, especially changes in the status of N limitation on vegetation under changing environment^8–10^. Vegetation N limitation is characterized as the lower supply of N to plants relative to their N demand and jointly determined by the provision of available N and plant N requirement^11–13^. In general, the greater the vegetation N limitation is, the less C vegetation would sequester and the more likely permafrost ecosystems function as a C source; on the contrary, permafrost ecosystems would tend to act as a C sink^8,14^. Consistent with this conceptual understanding, ecosystem C stock simulated with a C–N model configuration could be ~35 Pg lower than that simulated with a C-only configuration across the circumpolar permafrost region in 2300^8^. Therefore, deeper exploration of ecosystem N dynamics in permafrost regions is imperative to better predict permafrost C cycle and its feedback to climate warming.

During the last decade, the global change research community has paid great attention to the site-level dynamics of available N supply to plants in permafrost ecosystems^15–20^. Based on these efforts, an enhanced supply of plant-available N derived from the accelerated N mineralization or the release of originally frozen available N is observed with climate warming or permafrost thaw^15–20^. In addition, there are also studies that reveal increased plant productivity^17,21^ or enhanced plant N pool^17^ in permafrost ecosystems, reflecting an elevated plant N demand under environmental changes. These studies have advanced our understanding on both the dynamics of available N supply and plant N demand in permafrost regions. However, little is known about the dynamics of N supply relative to plant N demand across permafrost regions, especially over the broad geographic scale. This knowledge gap constrains our understanding on the status of vegetation N limitation and prevents an accurate prediction of the direction and magnitude of permafrost C-climate feedback.

**Fig. 1:**
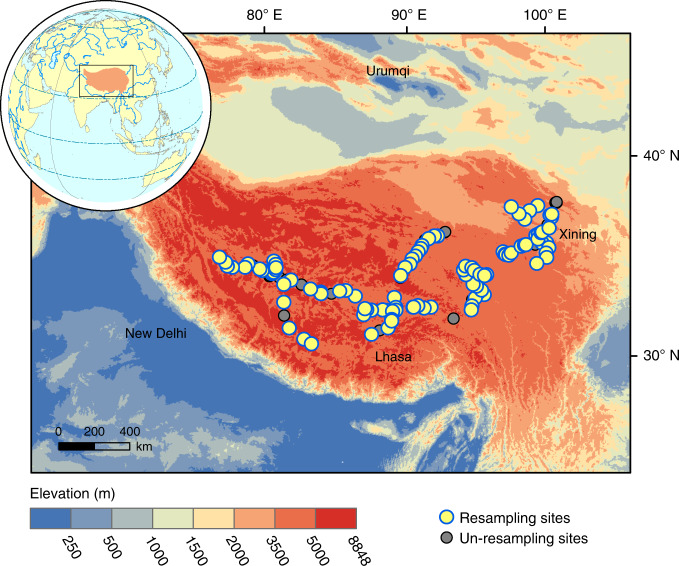
Distribution of sampling sites on the Tibetan Plateau. Yellow and grey circles jointly represent the original 135 sampling sites surveyed during the period of 2001 ~ 2004. Among them, the yellow circles denote the 107 resampling sites investigated during 2013~2014 and the grey circles indicate the unresampling sites due to practical limits such as road rebuilding. The background map represents the elevation across the study area.

To fill the knowledge gap mentioned above, we conducted a large-scale repeated sampling during the 2000s and the 2010s along a ~3000 km transect on the Tibetan Plateau (Fig. 1 and Supplementary Fig. 1), the largest alpine permafrost region around the world^22^, which has experienced fast warming, elevated atmospheric CO_2_ concentration (Supplementary Fig. 2) and extensive permafrost thaw as other permafrost regions^4,23^. The initial field campaign that investigated 135 sites across the study area was carried out during the period 2001~2004, while the revisited field campaign that covered 107 of the original 135 sites was performed during the period of 2013~2014 (Fig. 1). For each paired sampling sites, the investigation methods are identical (see details in “Methods” section). Based on the collected samples during the two sampling campaigns, plant δ^15^N and bulk soil δ^15^N (i.e., δ^15^N of total inorganic and organic N pools in the soil) were measured to characterize changes in ecosystem N cycle in this alpine permafrost region. To further explore ecosystem N dynamics, N cycling processes were simulated for each resampling site with a process-based biogeochemical model—DeNitrification-DeComposition (DNDC)^24,25^. With all the obtained measurements and simulations, this study aims to examine the temporal changes in ecosystem N cycle, especially vegetation N limitation across the Tibetan alpine permafrost region over the last decade. We find that plant growth becomes more N-limited in this alpine permafrost region despite the enhanced provision of plant-available N.

## Results and discussion

### Decreased plant δ^15^N over time

**Fig. 2:**
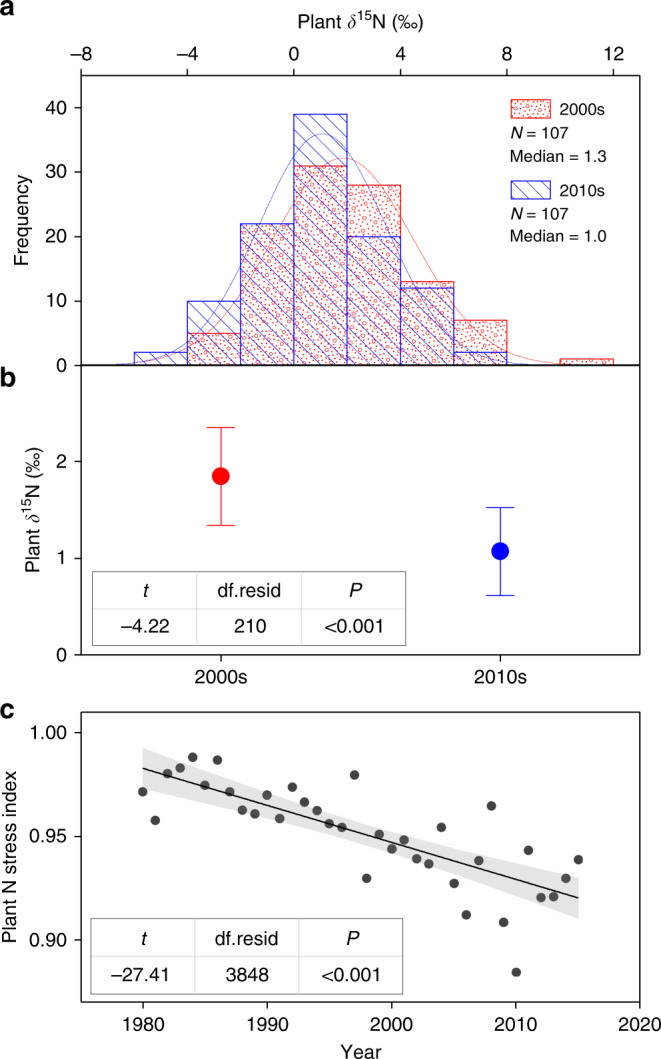
Changes in plant δ^15^N and N stress index over time. **a**–**c** Frequency distributions of plant δ^15^N during the two sampling periods, changes in plant δ^15^N derived from the large-scale resampling investigations and changes in plant N stress index derived from the DeNitrification-DeComposition (DNDC) model, respectively. Plant N stress index refers to the supply of N relative to plant demand, with a lower value meaning a greater limitation of N on plant growth^38–40^. Temporal dynamics of both indicators were examined with linear mixed-effects models, in which the fixed effect was year and the random effect was sampling site. Points in **b** denote mean values and error bars represent 95% confidence intervals. The shade accompanying with the solid fitted line in **c** represents 95% confidential interval. *N*, the number of sites used for analyzing plant δ^15^N; median, median value of plant δ^15^N; df.resid, residual degrees of freedom.

Over the past decade, neither mean annual precipitation (MAP; Supplementary Fig. 2) nor atmospheric N deposition (Supplementary Figs. 2 and 3) experienced significant changes, whereas both mean annual air temperature (MAAT) and atmospheric CO_2_ concentration significantly increased (*P* < 0.05; Supplementary Fig. 2) across the Tibetan alpine permafrost region. Under these environmental changes, plant δ^15^N across this alpine permafrost region significantly decreased during the period from the 2000s to the 2010s (median in the 2000s: 1.3‰, median in the 2010s: 1.0‰, *P* < 0.001; Fig. 2a, b). On average, the absolute changing rate in plant δ^15^N was −0.02‰ per year (within the range of −0.043 ~ −0.016‰ per year globally^13^) and the relative changing rate was −1.7% per year. Further analyses revealed that plant δ^15^N tended to decrease among 73 of the total 107 resampling sites and the decline in plant δ^15^N remained significant until >74 resampling sites were removed from the initial analysis with 107 resampling sites (Supplementary Fig. 4), demonstrating the robustness of isotopic dynamics observed in this study.

**Fig. 3:**
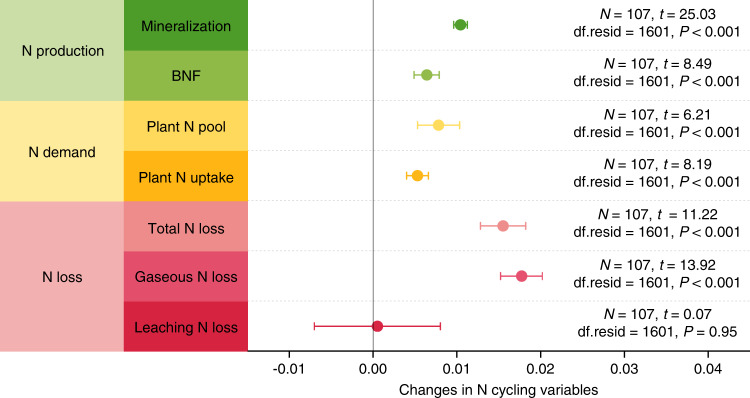
Temporal dynamics of N production, demand and loss. Changes in production of available N are reflected by annual soil N mineralization and biological N fixation rates (BNF), changes in plant N demand are represented by plant N pool and annual plant N uptake rate, and changes in ecosystem N loss are indicated by gaseous N loss, leaching N loss and total N loss (sum of gaseous and leaching N losses) over 2000s~2010s. Data of the N cycling variables were derived from the DeNitrification-DeComposition (DNDC) simulations. Changes in N cycling variables were characterized by the slope of relationship between an indicator and the fixed effect (year), which were examined with linear mixed-effects models after data normalization. Points in the plot denote the estimated model slopes and error bars represent 95% confidence intervals. *N*, the number of sites used for analyzing temporal dynamics of each indicator; df.resid, residual degrees of freedom.

**Fig. 4:**
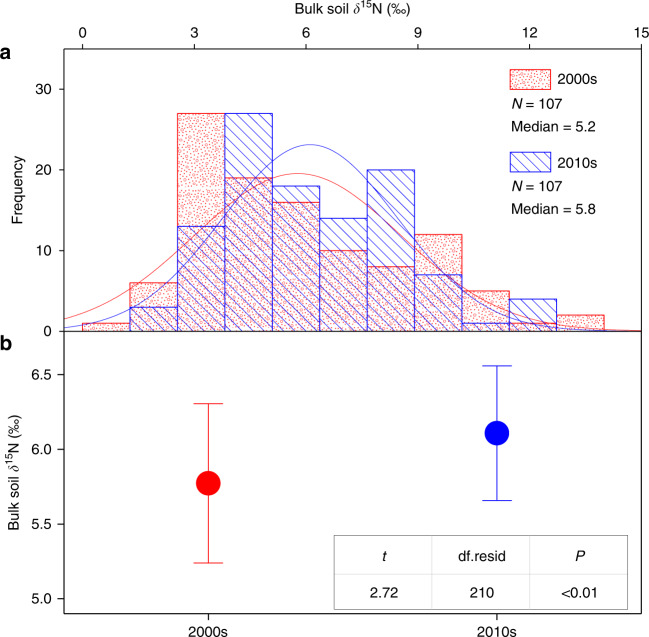
Changes in topsoil δ^15^N over 2000s ~ 2010s. **a**, **b** Frequency distributions of bulk soil δ^15^N and changes in bulk soil δ^15^N during the period between the 2000s and the 2010s, respectively. The change in bulk soil δ^15^N was examined with linear mixed-effects model, in which the fixed effect was year and the random effect was sampling site. Points in **b** denote mean values and error bars represent 95% confidence intervals. *N*, the number of sites used for analyzing bulk soil δ^15^N; Median, median value of bulk soil δ^15^N; df.resid, residual degrees of freedom.

Changes in plant δ^15^N could be associated with multiple reasons, such as shifts in vegetation community, atmospheric N deposition, biological N fixation and plant N uptake through root or mycorrhizae^26–29^. However, both vegetation community (Supplementary Figs. 5–7 and Supplementary Note 1) and atmospheric N deposition (both rate and δ^15^N; Supplementary Figs. 2 and 3) were stabilized over the two sampling periods, and thus had limited effects on the plant δ^15^N dynamics observed in this study. Moreover, despite biological N fixation increased over the last decade (*P* < 0.001; Fig. 3), it should not be the main reason for the decrease in plant δ^15^N, as plant δ^15^N also significantly decreased among the investigated sites without *Leguminosae* (*P* < 0.05; Supplementary Fig. 8). In addition, plant N uptake through roots could elevate rather than diminish plant δ^15^N, because bulk soil δ^15^N increased over the detection period (*P* < 0.01; Fig. 4) and the fractionation of N isotopes was minimal during root N uptake in natural ecosystems^29–31^. In contrast to root N uptake, plant N uptake through mycorrhizae can decrease plant δ^15^N given: (i) the transfer of ^15^N-deleted N to plants by mycorrhizae^13,26,28,29^; (ii) the crucial role of mycorrhizae in supplying N to plants under N-limited conditions^13,28,31,32^; (iii) the widespread distribution of plants associated with mycorrhizae^33^ across the 107 resampling sites; (iv) the potentially enhanced mycorrhizal colonization (Supplementary Fig. 9 and Supplementary Note 2) driven by the two major environmental changes on the Tibetan Plateau (CO_2_ enrichment and climate warming, Supplementary Fig. 2). Nevertheless, besides N limitation, other factors especially phosphorus (P) limitation and water stress could also make plants to invest more to mycorrhizae^31,34,35^. However, soil N : P ratio in the top 10 cm across the study area significantly decreased over the detection period (*P* < 0.01; Supplementary Fig. 10), indicating that N rather than P limitation was more likely to reduce plant δ^15^N via mycorrhizae. Moreover, topsoil moisture across the study area did not exhibit significant change over the detection period (Supplementary Figs. 11 and 12, and Supplementary Note 3), reflecting the minimal effect of water stress on the plant δ^15^N dynamics. Taken together, of all the factors considered, an enhancement in reliance on mycorrhizae for N acquisition could be the plausible reason for the decreased plant δ^15^N observed in this study.

As a widely used indicator to reflect the supply of N relative to plant N demand, a lower plant δ^15^N generally represents an elevated N limitation^12,13,26,27,36,37^. Due to this point, the decreased plant δ^15^N observed in this study suggests enhanced vegetation N limitation across the Tibetan alpine permafrost region over the last decade. To further demonstrate this point, we conducted a historical simulation from 1980 to 2015 for the 107 resampling sites with the DNDC model and acquired an indicator (plant N stress index) that represented the supply of N relative to plant demand^38–40^. The lower the plant N stress value is, the greater the vegetation N limitation is^38–40^. Our results showed that the plant N stress index significantly decreased during the past decades (*P* < 0.001; Fig. 2c), confirming the enhancement of vegetation N limitation across the study area. Besides the plant N stress index, we also explored the dynamics of available N production by analyzing changes in the simulated annual rates of soil N mineralization and biological N fixation. In contrast to the plant N stress index, these two processes were significantly accelerated during the period between the 2000s and the 2010s (*P* < 0.001; Fig. 3), indicating the enhanced production of available N across the study area. Collectively, these results highlighted that vegetation N limitation became stronger across the Tibetan alpine permafrost region during the past decade despite the increased production of available N.

### Enhanced plant N demand under changing environment

The enhancement of vegetation N limitation could be associated with increases in plant N demand^41^. To illustrate this point, we detected the changes in indicators characterizing plant N demand derived from the DNDC simulations, including plant N pool and annual plant N uptake rate. Results showed that both indicators significantly increased during the last decade (*P* < 0.001; Fig. 3), indicating that increased plant N demand could promote vegetation N limitation across the Tibetan alpine permafrost region. Based on the factorial analysis conducted through the DNDC model, we further disentangled the effects of environmental changes on plant N pool and annual plant N uptake rate. Our results illustrated that climate warming and CO_2_ enrichment played dominant roles in elevating these two parameters, highlighting their crucial effects on stimulating plant N demand (Supplementary Fig. 13a–d). Of them, climate warming can enhance vegetation productivity and subsequent plant N demand by accelerating plant metabolic activity and extending growing season length^36^, while CO_2_ enrichment can improve plant N demand through its fertilization effect^11,42^. In support of this interpretation, large amounts of evidences have revealed earlier growing season^43,44^, larger vegetation productivity^43,45^ and continuous ecosystem C sink^45,46^ across the Tibetan alpine permafrost region during the past decades.

### Increased ecosystem N loss induced by environmental change

The enhancement of vegetation N limitation could also be linked with processes that tend to decrease the supply of available N to plant growth^41^. To demonstrate this point, we examined changes in ecosystem N loss (gaseous N loss and leaching N loss) derived from the DNDC simulations. Our results showed that annual total ecosystem N loss rate significantly increased during the period from the 2000s to the 2010s (*P* < 0.001; Fig. 3) and the enhancement in total ecosystem N loss was largely due to the significant increase in gaseous N loss (*P* < 0.001; Fig. 3). The increase in gaseous N loss has potentials to reduce the supply of N relative to plant N demand and hence tends to induce the enhanced vegetation N limitation. Based on the factorial analysis conducted in the DNDC model, we found that climate warming was the major driver of the increased gaseous N loss over the past decade (Supplementary Fig. 13e, f). Ecologically, climate warming can promote gaseous N loss through (i) enhancing the production of available N substrates^47,48^ for nitrification and denitrification by stimulating microbial biomass or activity^49,50^, (ii) improving the supply of C for nitrifiers and denitrifiers by increasing root exudations^24,25^ and (iii) accelerating the diffusion of gaseous N generated by nitrification and denitrification processes^51^. Moreover, CO_2_ enrichment can also stimulate gaseous N loss (Supplementary Fig. 13e, f), probably due to the enhancement in soil biological activity or labile C availability under elevated CO_2_^52^. Besides climate warming and CO_2_ enrichment, soil moisture is known as another key factor regulating ecosystem gaseous N loss^47^, however, topsoil moisture across the study area experienced no significant change over the detection period (Supplementary Figs. 11 and 12, and Supplementary Note 3) and did not exhibit significant association with the simulated gaseous N loss (Supplementary Fig. 14 and Supplementary Note 3), indicating its limited effect on the increased gaseous N loss across the study area.

### Elevated bulk soil δ^15^N between the sampling interval

The increase in both plant N demand and ecosystem external N loss was confirmed by the increases in bulk soil δ^15^N observed in this study, which was a comprehensive indicator of ecosystem N cycle^29,53,54^. To be specific, our results showed that bulk soil δ^15^N in the top 10 cm across the Tibetan alpine permafrost region significantly increased during the period 2000s~2010s (median in the 2000s: 5.2‰, median in the 2010s: 5.8‰, *P* < 0.01; Fig. 4). The absolute changing rate of bulk soil δ^15^N was 0.05‰ per year and the relative changing rate was 0.9% per year. Bulk soil δ^15^N is determined by multiple N cycling processes, including gaseous N loss, net plant N uptake, leaching N loss, atmospheric N deposition and biological N fixation^53,54^. These processes have different effects on bulk soil δ^15^N, with the largest isotopic fractionation effects occurring in gaseous N loss (16~30‰), the second in net plant N uptake (5~10‰), the third in leaching N loss (1‰), and the least in atmospheric N deposition and biological N fixation (−2~0‰)^53,54^. Among these processes, atmospheric N deposition (both rate and δ^15^N, Supplementary Figs. 2–3) and leaching N loss (Fig. 3) did not exhibit significant changes during the detection period. Consequently, these two processes are supposed to have limited effects on bulk soil δ^15^N dynamics. Moreover, biological N fixation significantly increased during the past decade (*P* < 0.001; Fig. 3), which tended to depress bulk soil δ^15^N values because of the lower δ^15^N in biologically fixed N sources^53,54^. Accordingly, the increased bulk soil δ^15^N observed across the Tibetan alpine permafrost region should be largely driven by the enhanced gaseous N loss and/or the elevated plant N uptake. Overall, the simulated changes in plant N demand and ecosystem N loss (Fig. 3) were supported by the observed bulk soil δ^15^N dynamics (Fig. 4).

The progressive N limitation observed across the Tibetan alpine permafrost region contrasted with the traditional view that warming-induced permafrost thaw could eliminate vegetation N limitation in the circumpolar permafrost region by producing large amounts of available N through the acceleration of soil N mineralization and the release of originally frozen available N^15,17,19^. Such a difference could be related to the following two aspects. First, the soil N density (N storage per area) across the Tibetan alpine permafrost region (1.6 kg N m^−2^ to 3 m depth^55^) is much lower than that in the circumpolar permafrost region (4.6 ~ 7.5 kg N m^−2^ to 3 m depth^56^), which can lead to a lower available N supply through mineralization and thus is more likely to induce the enhanced vegetation N limitation. Second, the active layer thickness across the Tibetan alpine permafrost region (~2.4 m^23^) is two times deeper than that in the circumpolar permafrost region (~0.9 m^57^). Given that over 90% of plant roots are distributed within the 30 cm soil layer^58^, the deeper active layer thickness in this alpine permafrost region can restrict plants to use the released available N after permafrost thaw and thus is more likely to result in the enhanced vegetation N limitation.

In summary, based on isotopic observations from a resampling field investigation and simulations from a process-based biogeochemical model, this study explored ecosystem N dynamics across the Tibetan alpine permafrost region. We found an enhanced vegetation N limitation across the study area over the last decade despite the increase in available N production. The progressive N limitation was associated with the joint enhancement in plant N demand and ecosystem N loss under current environmental changes, especially climate warming and CO_2_ enrichment. These results suggest that the enhanced N limitation may constrain the positive effects of climate warming and CO_2_ enrichment on vegetation productivity^8^. Despite that, microbial respiration can still be stimulated by the continuous climate warming^59^. Consequently, the Tibetan alpine permafrost region would turn from the current C sink^45^ to future C source with continuous environmental changes, which could then switch the feedback of C cycle to climate warming in an opposite direction.

## Methods

### Study area

This study was conducted on the Tibetan Plateau, the largest alpine permafrost region around the world, with an area of ~1.35 × 10^6^ km^2^ and a mean elevation over 4000 m^22,23^. Climate on this plateau is cold and dry^22^. The MAAT ranges between −4.1 and 7.4 °C, and the MAP varies from dozens of mm in the northwest of the plateau to ~700 mm in the southeast^60^. Due to the low temperature, permafrost is extensively developed and categorized as continuous, discontinuous, sporadic and isolated types across the plateau^22^. The mean active layer thickness was estimated at ~2.4 m (range: 1.3~4.6 m) along the Qinghai-Tibetan highway^23^. Alpine grassland is the main vegetation type across the Tibetan alpine permafrost region, with dominant species being *Stipa purpurea* and *Carex moorcroftii* for alpine steppe and *Kobresia pygmaea* and *Kobresia humilis* for alpine meadow^45^. Based on the World Reference Base for Soil Resources classification, the soil type across the study area is dominated by Xerosols and Cambisols^45^.

During the past decade, atmospheric N deposition (Supplementary Figs. 2 and 3), MAP (Supplementary Fig. 2) and soil moisture in the top 10 cm (Supplementary Figs. 11 and 12, and Supplementary Note 3) kept stable across the Tibetan alpine permafrost region. Despite that, MAAT on the plateau has significantly increased with a rate of 0.05 °C per year since 1980^45^, which is similar to the rate reported in the circumpolar permafrost region and twice as much as the global mean warming rate^4^. As air temperature rose, soil temperature was also detected to increase continuously in this alpine permafrost region^23,45^. Warming climate has induced extensive permafrost thaw, including top-down permafrost degradation and various thermokarst features, such as thermo-erosion gullies and thermokarst lakes^23^. In addition, the Tibetan alpine permafrost region has also experienced a linearly increased atmospheric CO_2_ concentration at a rate of 2.2 p.p.m.v. per year (Supplementary Fig. 2). These environmental changes make the Tibetan alpine permafrost region to be a natural laboratory to explore ecosystem N dynamics.

### Original and repeated sampling

To detect temporal dynamics of ecosystem N cycle in the context of environmental changes across the Tibetan alpine permafrost region, a resampling field investigation, including an original field campaign and a repeated field campaign, was carried out across the study area during 2000s ~ 2010s (Fig. 1). To be specific, the original sampling campaign was conducted by the investigation team from Peking University during the period from 2001 to 2004^61^. Based on this original campaign, 135 sites were investigated across the plateau (Fig. 1). After about 10 years, the resampling field campaign was performed by the investigation team from Institute of Botany, Chinese Academy of Sciences in the year 2013 and 2014^45^. With the record of geographic location (latitude and longitude) of a historical site investigated during the 2000s, the resampling site was preliminarily determined by a Global Position System with a decimetre precision (Supplementary Fig. 1a). Based on the soil pits excavated during the 2000s, the resampling site was then accurately located with the help of investigators who participated in the original campaign during the 2000s (Supplementary Fig. 1b). Eventually, 107 (~79%) of the original 135 sites were re-investigated (Fig. 1). These sites spanned about ~3000 km on the Tibetan Plateau, with longitude ranging from 80.8 to 120.1°E and latitude varying between 29.3 and 49.5°N (Fig. 1). Moreover, these resampling sites covered a wide range of climate gradients (MAAT: −3.1 ~ 4.4 °C; MAP: 103 ~ 694 mm) and major vegetation types (59 sites in alpine steppe and 48 sites in alpine meadow) across the study area.

Vegetation samples were collected in the same manner during the two sampling periods. Specifically, in the initial field campaign, a plot of 10 m × 10 m was set up after locating a site and then five quadrats (1 m × 1 m) were established at each corner and the centre of the plot. Within the five quadrats, vegetation communities (occurred species and the relative cover) were investigated and aboveground vegetation was collected for plant δ^15^N analysis. In the repeated field survey, once the original 10 m × 10 m plot was recovered (Supplementary Fig. 1b), we set five 1 m × 1 m quadrats next to the original quadrats, investigated vegetation communities (occurred species and the relative cover) and collected all the aboveground vegetation in each of the five quadrats for plant δ^15^N analysis (Supplementary Fig. 1c). Consequently, the plant δ^15^N measured in our study represented the average isotopic signal of the vegetation community in the five quadrats with 1 × 1 m^2^ area. In other words, the isotopic signal represented information of all the plant species within the vegetation community. Considering the potential effects of shifts in vegetation community on plant δ^15^N dynamics observed in this study, we examined changes in vegetation composition indicated by species richness and Shannon–Wiener index (Supplementary Note 1) and detected the stable vegetation community over the detection period (Supplementary Fig. 5). Due to the potential effects of heavily mycorrhizal plant on the community average δ^15^N, we further analysed changes in species richness and the relative cover of five families that could be heavily colonized by mycorrhizae on the Tibetan Plateau (*Gramineae*, *Leguminosae*, *Asteraceae*, *Cyperaceae* and *Rosaceae*)^33^, and examined changes in the relative cover of the dominant species within the five families^33^ (Supplementary Note 1). Both the family- and species-level analyses confirmed that vegetation community composition was unchanged over the period from the 2000s to the 2010s (Supplementary Figs. 6 and 7), which eliminated the potential confounding effects of vegetation community shifts on the plant δ^15^N dynamics observed in this study.

Similar to vegetation samples, the corresponding approaches used to collect soil samples were also consistent between the two sampling periods. Specifically, soil samples used to measure N isotope, N content as well as other soil properties (texture, pH, soil organic carbon (SOC) content) were collected within the top 10 cm layer in three quadrats along a diagonal within each resampling site (Supplementary Fig. 1c). Soil samples used to determine bulk density were collected within the 10 cm depth with standard 100 cm^3^ steel cylinders in three quadrats along a diagonal within each resampling site^45^. Besides the identical investigation method and the stable vegetation community (Supplementary Figs. 5–7), the topography and management practices were also unchanged during the detection period for each of the 107 paired sampling sites^45^.

### Elemental and stable isotope analyses

To explore changes in ecosystem N cycle, we measured δ^15^N values of plant and bulk soil with samples collected through the large-scale resampling investigations. In our study, site-level plant δ^15^N measurement was conducted based on the five vegetation samples collected over the five quadrats (1 m × 1 m) within a sampling plot (10 m × 10 m; Supplementary Fig. 1). Specifically, at each sampling site, plant samples were collected from each of the five 1 × 1 m^2^ quadrats, dried to a constant weight at 65 °C and weighed as aboveground biomass separately. The dried plant samples were then ground using a ball mill (Mixer Mill MM 400, Retsch, Germany) for plant δ^15^N analysis. In addition, the collected soil samples were air dried, passed through a 2 mm sieve (removing gravel and coarse roots), processed to remove fine roots and ground with a ball mill (Mixer Mill MM 400, Retsch, Germany). With the treated plant and soil samples, we measured plant and bulk soil δ^15^N values using an isotope ratio mass spectrometer (SerCon 20–22, Crewe, UK), analysed plant and soil N contents with an elemental analyser (Vario EL Ш, Elementar, Germany), and determined soil P contents with an inductively coupled plasma optical emission spectrometry (ICAP 6300 ICP-OES Spectrometer, Thermo Scientific, USA). To avoid errors in measurements, both δ^15^N values and element contents during the two sampling periods were determined at the same time with the same instruments.

### Model simulation

To further examine ecosystem N dynamics on the basis of observations from large-scale resampling investigation, we simulated the key ecosystem N cycling processes for the 107 resampling sites with DNDC [http://www.dndc.sr.unh.edu/], a process-based biogeochemical model. DNDC has been widely tested and applied around the world [http://www.globaldndc.net/information/publications-i-3.html], especially for the simulation of ecosystem N dynamics^24,25^, and has also been demonstrated to perform well across the Tibetan alpine permafrost region^62,63^.

In this study, we first validated the DNDC model in terms of simulating ecosystem-atmosphere nitrous oxide (N_2_O) fluxes. Specifically, we collected N_2_O fluxes measurements from published studies across the Tibetan alpine permafrost region, and obtained 58 N_2_O observations from 22 sites (Supplementary Fig. 15a) in 18 studies (Supplementary Methods). We also synthesized other data from the above-mentioned literature (Supplementary Methods) and other associated literature^64–67^ to drive the model simulation, including vegetation type, plant biomass, plant C:N ratio, soil texture, soil bulk density, soil pH, SOC content and soil C:N ratio. To drive the model simulation, we further acquired data for atmospheric CO_2_ concentration, annual rate of atmospheric N deposition, daily meteorology and thermal degree days. Among them, the atmospheric CO_2_ concentration, observed at the Waliguan (a global atmospheric background station), was obtained from the Chinese Research Network or Special Environment and Disaster [http://www.crensed.ac.cn]. Atmospheric N deposition rate was obtained from a national-scale spatiotemporal dataset^68^ if it was not provided in the original literature. The daily meteorological data, including mean temperature, maximum temperature, minimum temperature and precipitation, were derived from the nearest meteorological station [http://data.cma.cn/] close to the N_2_O observation site. The thermal degree days were calculated by summing the daily mean temperature higher than 0 °C^38^. Based on the above-mentioned dataset, N_2_O fluxes were simulated by the DNDC model. With the simulated and observed N_2_O emissions, the model performance was evaluated on the basis of the 1 : 1 line plot (Supplementary Fig. 15b) and four indicators which included *R*^2^ (coefficient of determination; Eq. 1), RMSE (root mean square error; Eq. 2), RMD (relative mean deviation; Eq. 3) and ME (model efficiency; Eq. 4)^62,63^.

1 $$R^2 = \frac{{\left[ {\mathop {\sum }\nolimits_{i = 1}^n {\left( {O_i - \bar O} \right)} {\left( {S_i - \bar S} \right)} } \right]^2}}{{\mathop {\sum }\nolimits_{i = 1}^n {\left( {O_i - \bar O} \right)} ^2\mathop {\sum }\nolimits_{i = 1}^n {\left( {S_i - \bar S} \right)} ^2}}$$

2 $${\mathrm{RMSE}} = \frac{{{\mathrm{100}}}}{{\bar O}}\sqrt {\frac{{\mathop {\sum }\nolimits_{i = 1}^n \left( {S_i - O_i} \right)^2}}{n}}$$

3 $${\mathrm{RMD}} = \frac{{{\mathrm{100}}}}{{\bar O}}\mathop {\sum }\limits_{i = 1}^n \frac{{S_i - O_i}}{n}$$

4 $${\mathrm{ME}} = 1 - \frac{{\mathop {\sum }\nolimits_{i = 1}^n (S_i - O_i)^2}}{{\mathop {\sum }\nolimits_{i = 1}^n (O_i - \bar O)^2}}$$

where in *S*_*i*_ and *O*_*i*_ are simulated and observed values, $\bar S$ and $\bar O$ are their averages and *n* indicates the number of simulation-observation pairs. The model validation showed that all the data points constituted by the simulated and observed N_2_O emissions were well distributed near the 1 : 1 line (Supplementary Fig. 15b), demonstrating good model performance across the study area.

Using the validated model, we conducted simulations for the 107 resampling sites during the period from 1980 to 2015. Before simulations, credible data were collected as model inputs, including atmospheric CO_2_ concentration, annual atmospheric N deposition rate, daily meteorological data, vegetation type, plant biomass, plant C : N ratio, soil texture, soil bulk density, soil pH, SOC content and soil C : N ratio. Among them, the annual atmospheric CO_2_ concentration, annual atmospheric N deposition rate and daily meteorological data were derived from the Chinese Research Network or Special Environment and Disaster [http://www.crensed.ac.cn], a spatiotemporal dataset of atmospheric N deposition in China^68^ and China Meteorological Administration [http://data.cma.cn/], respectively. The vegetation type for each site was determined based on the vegetation community investigation during the resampling campaign. Data for plant biomass, plant C : N ratio, soil texture, bulk density, pH, SOC content and soil C : N ratio were derived from direct measurements. Of them, plant C content was measured with an elemental analyser (Vario EL Ш, Elementar, Germany). Soil texture was determined with a laser particle size analyser (Malvern Masterizer 2000, Malvern, UK) after removing soil organic matter and carbonate. Soil bulk density was examined by drying the samples collected with standard steel cylinders at 105 °C. Soil pH was determined using a pH electrode (PB-10, Sartorius, Germany) in a 1 : 2.5 soil-to-deionized water mixture. The SOC content was analysed with the Walkley–Black method^61^. With the above-mentioned dataset, simulations were conducted with the DNDC model. After that, the model was further validated with the observed soil N density (N storage per area, 0–10 cm, Eq. 5) and aboveground plant N pool (the product of aboveground biomass and the corresponding N content), based on the combination of 1:1 line plots and the four model performance indicators mentioned above (i.e., *R*^2^, RMSE, RMD and ME). The validation results showed that both soil N density and aboveground plant N pool were well distributed near the 1 : 1 line and the four indicators (i.e., *R*^2^, RMSE, RMD, and ME) illustrated good model performance (Supplementary Fig. 16).

5 $${{SND}} = \mathop {\sum }\limits_{i = 1}^n T_i \times {{BD}}_i \times {{TN}}_i \times \frac{{\left( {1 - C_i} \right)}}{{{\mathrm{100}}}}$$

In Eq. 5, soil N density (*SND*, kg N m^−2^) was calculated based on soil thickness (*T*_*i*_, cm), bulk density (*BD*_*i*_, g cm^−3^), soil total N content (*TN*_*i*_, g kg^−1^) and >2 mm rock content (*C*_*i*_, %)^55^. In addition, soil moisture (10 cm depth) simulated by the DNDC model, determined jointly by precipitation and other hydrological processes^69^, was also validated with the measured values across the 107 resampling sites. The data-model comparison revealed that the DNDC model could well capture the observed soil moisture across the investigated sites (Supplementary Fig. 17). Furthermore, both the simulated and observed surface soil water-filled pore space experienced no significant changes over the period from the 2000s to the 2010s (Supplementary Figs. 11 and 12), demonstrating that the DNDC model could also accurately characterize soil moisture dynamics across the study area.

### Statistical analyses

To determine whether N cycling variables exhibited significant differences during the detection period, we conducted statistical tests with the linear mixed-effects model (LMM). LMM is an extension of simple linear model, which can effectively remove the random effects for non-independent data^45^. In this study, LMMs were used to explore changes in plant δ^15^N, plant N stress index, the production of available N (soil N mineralization and biological N fixation), plant N demand (plant N pool and plant N uptake rate), ecosystem N loss (total N loss, gaseous N loss and leaching N loss) and bulk soil δ^15^N over the detection period. For all the analyses with LMM, the fixed effect was year and the random effect was sampling site. Data normality was tested before the LMM analyses, and log-transformation was performed when necessary. All the analyses were conducted in R 3.5.1 with the lme4 package^70^.

To disentangle effects of environmental changes on N cycling processes, factorial analyses were conducted with the DNDC simulations. Four simulation experiments were carried out for each of the 107 resampling sites: (i) constant atmospheric CO_2_ concentration with normal changes in other factors; (ii) constant temperature with normal changes in other factors; (iii) constant precipitation with normal changes in other factors; and (iv) normal changes in all factors. After all the simulations, we calculated the differences between the target variable in one of the four controlling simulation experiments and the target variable in experiment (iv) for each resampling site. Finally, we averaged the calculated differences among the 107 resampling sites to reflect the impact of different environmental changes on the dynamics of N cycling processes.

## Supplementary information

Supplementary Information

Peer Review File

Supplementary Dataset 1

## Data Availability

All plant and soil δ^15^N data used in this study are available as a supplementary file (Supplementary Data 1). Additional data are available from the corresponding author (Dr. Yuanhe Yang) upon reasonable request.
