# Supplementary material for: Progressive nitrogen limitation across the Tibetan alpine permafrost region
Source: Nat Commun. 2020 Jul 3;11:3331. doi: 10.1038/s41467-020-17169-6 (PMC7335038; doi:10.1038/s41467-020-17169-6)
Supplement: Supplementary file 1 — Supplementary Information [file 41467_2020_17169_MOESM1_ESM.pdf]

Supplementary Information for

Progressive nitrogen limitation across the Tibetan alpine permafrost region

Kou et al.

Supplementary Notes

Supplementary Note 1. Analyses for changes in vegetation community between the two sampling periods

Vegetation communities were investigated for both periods of our resampling campaign. During the investigation, all the occurred plant species were identified, and the relative cover of each species was virtually estimated. Based on these information, two indexes including species richness and Shannon-Wiener index were calculated to characterize the vegetation community composition in each of the two sampling periods. Of them, species richness refers to the species number per quadrat, and Shannon-Wiener index (H) was calculated as: $H = -\sum P_i \ln P_i$, where P_i is the relative cover of species i[1]. With the calculated species richness and Shannon-Wiener index in the 2000s and the 2010s, we examined changes in vegetation composition between the two sampling periods based on linear mixed-effects models, in which the fixed effect was sampling years and the random effects were the investigated sites as well as replicates within each site[2]. Results showed that neither of the two indicators exhibited any significant changes over the detection period (Supplementary Fig. 5), indicating the minimal effect of vegetation community shifts on the dynamics of plant $\delta^{15}N$ observed in this study. Considering the potential effects of mycorrhizal plants on the average community $\delta^{15}N$, we further detected changes in vegetation composition at the family and species levels that are associated with mycorrhizae. Of them, changes in vegetation composition at the family level were analyzed based on two indicators (species richness and the relative cover) for five families that can be heavily colonized by mycorrhizae on the Tibetan Plateau, including *Gramineae*, *Leguminosae*, *Asteraceae*, *Cyperaceae* and *Rosaceae*[3]. The species-level analyses were conducted to detect changes in the relative cover for the dominant species of the five families mentioned above. With linear mixed-effect

models, results also indicated that the vegetation composition was unchanged either at the family or species level between the 2000s and the 2010s (Supplementary Figs. 6-7). Taken together, these analyses eliminated the potential confounding effects of vegetation community shifts on the plant $\delta^{15}N$ dynamics observed in this study.

Supplementary Note 2. Meta-analyses of CO_2 enrichment and warming effects on mycorrhizal symbiosis

Meta-analysis was conducted to explore the potential effects of two major environmental changes on the Tibetan Plateau, i.e., elevated CO_2 (eCO_2) and climate warming on mycorrhizal symbiosis. To be specific, by searching with mycorrhiza* and CO_2 or mycorrhiza* and carbon dioxide in the Web of Science, we collected studies that reported the responses of mycorrhizal symbiosis to eCO_2. Likewise, we synthesized publications that investigated the effects of experimental warming on mycorrhizal symbiosis with terms mycorrhiza* and warming or mycorrhiza* and temperature. All collected publications dated to February 25th, 2020. To avoid biases, the following criteria were adopted during the searching process: (i) variables that represented mycorrhizal symbiosis (e.g., percent of root length colonized and extraradical mycorrhizal hyphal density) were reported; (ii) mean value, sample size and error (standard error, standard deviation or confidence interval) for eCO_2 or warming were recorded or could be calculated from the individual studies; (iii) treatment (eCO_2 or warming) and control plots had same ecosystem types, soil types and dominant plant species. Finally, two datasets were compiled including 100 studies that reported eCO_2 effects on mycorrhizal symbiosis and 22 studies that recorded warming effects on mycorrhizal symbiosis.

As commonly used in meta-analysis, we adopted the response ratio (RR) to quantify the responses of mycorrhizal symbiosis to eCO_2 or experimental warming. The RR refers to the natural-logarithm-transformed ratio of target variables associated with mycorrhizal symbiosis between experimental treatment and control[4]. Typically, the collected data are weighted either by variance or sample size, and data with a lower variance or larger sample size are assumed to contribute more to the mean effect size. Because the collected studies did not always report the sampling variance (e.g., the standard error or standard deviation), the number of replicates was used as the weighting factor, i.e., $weight_n = n_c n_t/(n_c+n_t)$, where n_c and n_t are the number of replicates for control and treatment groups, respectively[5]. A random-effect model with bootstrap approach was applied to estimate the mean effect size and its 95% confidence interval (CI) with MetaWin 2.1 (Sinauer Associates Inc., Sunderland, MA, USA). The eCO_2 or warming effect was considered significant if the 95% CI did not overlap with zero. Based on the meta-analyses, we observed that both eCO_2 and climate warming significantly enhanced the degree of mycorrhizal colonization ($P < 0.05$, Supplementary Fig. 9).

Supplementary Note 3. Changes in soil moisture on the Tibetan Plateau during 2000s-2010s and its potential effects on gaseous N loss

We collected four independent data sources to examine soil moisture dynamics in the Tibetan alpine permafrost region during 2000s-2010s. The first data source was derived from our resampling investigations. Based on this dataset, we analyzed changes in the gravimetric soil moisture in the top 10 cm with the linear mixed-effects model. Results showed that surface soil moisture did not exhibit any significant differences between the two sampling periods (Supplementary Fig. 11). The second data source was

purchased from China Meteorological Administration (http://data.cma.cn/). Using field observations recorded by 7 meteorological stations across the study area, we found that the average surface (0-10 cm) soil moisture did not exhibit any significant trend over 2000-2010 (Supplementary Fig. 12a). The third data source was derived from a recent study exploring permafrost changes and their effects on hydrological processes on the Tibetan Plateau, which provided the observed dataset for soil moisture dynamics at five monitoring sites in the hinterland of the Tibetan alpine permafrost region[6]. By averaging the monitored data, we observed that the surface soil moisture remained stable over the detection period (Supplementary Fig. 12b). The fourth data source was obtained from a global dataset, CFSV2 (NCEP Climate Forecast System Version 2)[7]. From the CFSV2 dataset, we extracted the surface (5 cm) volumetric soil moisture for the 107 resampling sites, and found that the surface soil moisture on the Tibetan Plateau experienced no significant changes over 2000-2015 (Supplementary Fig. 12c). Collectively, results from these four independent data sources consistently demonstrated that the relatively stable surface soil moisture on the Tibetan Plateau from the 2000s to the 2010s. The stability of soil moisture indicated its limited effect on the increase in gaseous N loss observed across the study area. To further demonstrate this point, we explored the relationship between each year's total gaseous N loss from denitrification with the corresponding annual average soil water-filled pore space over the detection period. Results showed that this relationship was insignificant (Supplementary Fig. 14), which provided additional evidence for the argument that soil moisture dynamics could not be responsible for the increased gaseous N loss across our study area.

Supplementary Methods

Supplementary Methods: References used to synthesize N_2O observations for DNDC model validation

1. Cai, Y. J. et al. The impact of excretal returns from yak and Tibetan sheep dung on nitrous oxide emissions in an alpine steppe on the Qinghai-Tibetan Plateau. *Soil Biol. Biochem.* **76**, 90-99 (2014).
2. Chen, X. et al. Effects of warming and nitrogen fertilization on GHG flux in the permafrost region of an alpine meadow. *Atmos. Environ.* **157**, 111-124 (2017).
3. Fu, Y. F. et al. Quantification of year-round methane and nitrous oxide fluxes in a typical alpine shrub meadow on the Qinghai-Tibetan Plateau. *Agric. Ecosyst. Environ.* **255**, 27-36 (2018).
4. Hu, Y. et al. Effects of warming and grazing on N_2O fluxes in an alpine meadow ecosystem on the Tibetan plateau. *Soil Biol. Biochem.* **42**, 944-952 (2010).
5. Jiang, C., Yu, G., Fang, H., Cao, G. & Li, Y. Short-term effect of increasing nitrogen deposition on CO_2, CH_4 and N_2O fluxes in an alpine meadow on the Qinghai-Tibetan Plateau, China. *Atmos. Environ.* **44**, 2920-2926 (2010).
6. Li, Y. Y. et al. Seasonal changes of CO_2, CH_4 and N_2O fluxes in different types of alpine grassland in the Qinghai-Tibetan Plateau of China. *Soil Biol. Biochem.* **80**, 306-314 (2015).
7. Pei, Z., Ouyang, H., Zhou, C. & Xu, X. Fluxes of CO_2, CH_4 and N_2O from alpine grassland in the Tibetan Plateau. *J. Geograph. Sci.* **13**, 27-34 (2003).
8. Peng, Y. F. et al. Soil temperature dynamics modulate N_2O flux response to multiple nitrogen additions in an alpine steppe. *J. Geophys. Res. Biogeo.* **123**, 3308-3319 (2018).
9. Liu, Y. W. et al. Plant and soil responses of an alpine steppe on the Tibetan

Plateau to multi-level nitrogen addition. *Plant Soil* **373**, 515-529 (2013).

10. Wang, G. S. et al. Greenhouse Gas Emissions from the Tibetan Alpine Grassland: Effects of Nitrogen and Phosphorus Addition. *Sustainability* **10**, (2018).

11. Wei, D. et al. Responses of CO_2, CH_4 and N_2O fluxes to livestock exclosure in an alpine steppe on the Tibetan Plateau, China. *Plant Soil* **359**, 45-55 (2012).

12. Wei, D., Xu, R., Liu, Y., Wang, Y. & Wang, Y. Three-year study of CO_2 efflux and CH_4/N_2O fluxes at an alpine steppe site on the central Tibetan Plateau and their responses to simulated N deposition. *Geoderma* **232**, 88-96 (2014).

13. Yan, Y. L. et al. Nitrogen deposition induced significant increase of N_2O emissions in an dry alpine meadow on the central Qinghai-Tibetan Plateau. *Agric. Ecosyst. Environ.* **265**, 45-53 (2018).

14. Yao, Z. S. et al. Characteristics of annual greenhouse gas flux and NO release from alpine meadow and forest on the eastern Tibetan Plateau. *Agric. For. Meteorol.* **272**, 166-175 (2019).

15. Zhang, H. et al. Annual N_2O emissions from conventionally grazed typical alpine grass meadows in the eastern Qinghai-Tibetan Plateau. *Sci. Total Environ.* **625**, 885-899 (2018).

16. Zhang, W. et al. The increasing distribution area of zokor mounds weaken greenhouse gas uptakes by alpine meadows in the Qinghai-Tibetan Plateau. *Soil Biol. Biochem.* **71**, 105-112 (2014).

17. Zhang, Z. H. et al. Nitrous oxide emissions from different land uses affected by managements on the Qinghai-Tibetan Plateau. *Agric. For. Meteorol.* **246**, 133-141 (2017).

18. Zhu, X. X. et al. Effects of warming, grazing/cutting and nitrogen fertilization on greenhouse gas fluxes during growing seasons in an alpine meadow on the

Tibetan Plateau. *Agric. For. Meteorol.* **214**, 506-514 (2015).

Supplementary Methods: References used to analyze the effects of CO_2 enrichment on mycorrhiza symbiosis

1 Alberton, O. & Kuyper, T. W. Ectomycorrhizal fungi associated with Pinus sylvestris seedlings respond differently to increased carbon and nitrogen availability: implications for ecosystem responses to global change. *Glob. Chang. Biol.* **15**, 166-175 (2009).

2 Alberton, O., Kuyper, T. W. & Gorissen, A. Competition for nitrogen between *Pinus sylvestris* and ectomycorrhizal fungi generates potential for negative feedback under elevated CO_2. *Plant Soil* **296**, 159-172 (2007).

3 Alguacil, M. D. M., Kohler, J., Caravaca, F. & Roldán, A. Differential effects of *Pseudomonas mendocina* and *Glomus intraradices* on lettuce plants physiological response and aquaporin PIP2 gene expression under elevated atmospheric CO_2 and drought. *Microb. Ecol.* **58**, 942-951 (2009).

4 Arndal, M. F., Merrild, M. P., Michelsen, A., Schmidt, I. K., Mikkelsen, T. N. & Beier, C. Net root growth and nutrient acquisition in response to predicted climate change in two contrasting heathland species. *Plant Soil* **369**, 615-629 (2013).

5 Baslam, M., Antolín, M.C., Gogorcena, Y., Muñoz, F. & Goicoechea, N. Changes in alfalfa forage quality and stem carbohydrates induced by arbuscular mycorrhizal fungi and elevated atmospheric CO_2. *Ann. Appl. Biol.* **164**, 190-199 (2014).

6 Baslam, M., Erice, G. & Goicoechea, N. Impact of arbuscular mycorrhizal fungi (AMF) and atmospheric CO_2 concentration on the biomass production and partitioning in the forage legume alfalfa. *Symbiosis* **58**, 171-181 (2012).

7 Baslam, M., Garmendia, I. & Goicoechea, N. Elevated CO_2 may impair the

beneficial effect of arbuscular mycorrhizal fungi on the mineral and phytochemical quality of lettuce. *Ann. Appl. Biol.* **161**, 180-191 (2012).

8 Becklin, K. M., Mullinix, G. W. R. & Ward, J. K. Host Plant Physiol. and mycorrhizal functioning shift across a glacial through future [CO_2] gradient. *Plant Physiol.* **172**, 789-801 (2016).

9 Berntson, G. M. & Bazzaz, F. A. Regenerating temperate forest mesocosms in elevated CO_2: belowground growth and nitrogen cycling. *Oecologia* **113**, 115-125 (1998).

10 Berntson, G. M., Wayne, P. M. & Bazzaz, F. A. Below-ground architectural and mycorrhizal responses to elevated CO_2 in *Betula alleghaniensis* populations. *Funct. Ecol.* **11**, 684-695 (1997).

11 Bettoni, M. M., Mogor, Á. F., Pauletti, V. & Goicoechea, N. Growth and metabolism of onion seedlings as affected by the application of humic substances, mycorrhizal inoculation and elevated CO_2. *Sci. Hortic.* **180**, 227-235 (2014).

12 Bettoni, M. M., Mogor, Á. F., Pauletti, V. & Goicoechea, N. The Interaction between Mycorrhizal Inoculation, Humic Acids Supply and Elevated Atmospheric CO_2 Increases Energetic and Antioxidant Properties and Sweetness of Yellow Onion. *Hortic. Environ. Biotechnol.* **58**, 432-440 (2017).

13 Cavagnaro, T. R., Sokolow, S. K. & Jackson, L. E. Mycorrhizal effects on growth and nutrition of tomato under elevated atmospheric carbon dioxide. *Funct. Plant Biol.* **34**, 730-736 (2007).

14 Chen, X., Tu, C., Burton, M. G., Watson, D. M., Burkey, K. & Hu, S. Plant nitrogen acquisition and interactions under elevated carbon dioxide: impact of endophytes and mycorrhizae. *Glob. Chang. Biol.* **13**, 1238-1249 (2007).

15 Choi, D. S. et al. Mycorrhizal Activities in Pinus densiflora, *P. koraiensis* and *Larix kaempferi* Native to Korea Raised under High CO_2 Concentrations and Water Use Efficiency. *Phyton* **45**, 139-144 (2005).

16 Choi, D., Makoto, K., Quoreshi, A. M. & Qu, L. Seed germination and seedling physiology of *Larix kaempferi* and *Pinus densiflorain* seedbeds with charcoal and elevated CO_2. *Landsc. Ecol. Eng.* **5**, 107-113 (2009).

17 Clark, N. M., Rillig, M. C. & Nowak, R. S. Arbuscular mycorrhizal fungal abundance in the mojave desert: seasonal dynamics and impacts of elevated CO_2. *J. Arid Environ.* **73**, 834-843 (2009).

18 Delucia, E. H., Callaway, R. M., Thomas, E. M. & Schlesinger, W. H. Mechanisms of phosphorus acquisition for ponderosa pine seedlings under high CO_2, and temperature. *Ann. Bot.* **79**, 111-120 (1997).

19 Denef, K., Bubenheim, H., Lenhart, K., Vermeulen, J., Van Cleemput, O., Boeckx, P. & Müller, C. Community shifts and carbon translocation within metabolically-active rhizosphere microorganisms in grasslands under elevated CO_2. *Biogeosciences* **4**, 769-779 (2007).

20 Dhillion, S. S., Roy, J. & Abrams, M. Assessing the impact of elevated CO_2 on soil microbial activity in a Mediterranean model ecosystem. *Plant Soil* **187**, 333-342 (1996).

21 Fortuna, P., Avio, L., Morini, S. & Giovannetti, M. Fungal biomass production in response to elevated atmospheric CO_2 in a *Glomus mosseae-Prunus* cerasifera model system. *Mycol. Prog.* **11**, 17-26 (2012).

22 Fransson, P. M. A., Anderson, I. C. & Alexander, I. J. Does carbon partitioning in ectomycorrhizal pine seedlings under elevated CO_2 vary with fungal species? *Plant Soil* **291**, 323-333 (2007).

23 Fransson, P. M. A., Taylor, A. F. S. & Finlay, R. D. Mycelial production, spread and root colonisation by the ectomycorrhizal fungi *Hebeloma crustuliniforme* and *Paxillus involutus* under elevated atmospheric CO_2. *Mycorrhiza* **15**, 25-31 (2005).

24 Gamper, H., Peter, M., Jansa, J., Lüscher, A., Hartwig, U. & Leuchtmann, A. Arbuscular mycorrhizal fungi benefit from 7 years of free air CO_2 enrichment in well-fertilized grass and legume monocultures. *Glob. Chang. Biol.* **10**, 189-199 (2004).

25 Garcia, M. O., Ovasapyan, T., Greas, M. & Treseder, K. K. Mycorrhizal dynamics under elevated CO_2 and nitrogen fertilization in a warm temperate forest. *Plant Soil* **303**, 301-310 (2008).

26 Gavito, M. E., Bruhn, D. & Jakobsen, I. Phosphorus uptake by arbuscular mycorrhizal hyphae does not increase when the host plant grows under atmospheric CO_2 enrichment. *New Phytol.* **154**, 751-760 (2002).

27 Gavito, M. E., Curtis, P. S., Mikkelsen, T. N. & Jakobsen, I. Atmospheric CO_2 and mycorrhiza effects on biomass allocation and nutrient uptake of nodulated pea (*Pisum sativum* L.) plants. *J. Exp. Bot.* **51**, 1931-1938 (2000).

28 Gavito, M., Schweiger, P. & Jakobsen, I. P uptake by arbuscular mycorrhizal hyphae: effect of soil temperature and atmospheric CO_2 enrichment. *Glob. Chang. Biol.* **9**, 106-116 (2003).

29 Godbold, D. L., Berntson, G. M. & Bazzaz, F. A. Growth and mycorrhizal colonization of three North American tree species under elevated atmospheric CO_2. *New Phytol.* **137**, 433-440 (1997)

30 Goicoechea, N., Baslam, M., Erice, G. & Irigoyen, J. J. Increased photosynthetic acclimation in alfalfa associated with arbuscular mycorrhizal

fungi (AMF) and cultivated in greenhouse under elevated CO_2. *J. Plant Physiol.* **171**, 1774-1781 (2014).

31 Goicoechea, N., Bettoni, M. M., Fuertes-Mendizábal, T., González-Murua, C. & Aranjuelo, I. Durum wheat quality traits affected by mycorrhizal inoculation, water availability and atmospheric CO_2 concentration. *Crop Pasture Sci.* **67**, 147-155 (2016).

32 Gorissen, A. & Kuyper, T. W. Fungal species-specific responses of ectomycorrhizal Scots pine (*Pinus sylvestris*) to elevated [CO_2]. *New Phytol.* **146**, 163-168 (2000).

33 Gutknecht, J. M., Field, C. B. & Balser, T. Microbial communities and their responses to simulated global change fluctuate greatly over multiple years. *Glob. Chang. Biol.* **18**, 1-14 (2012).

34 Hartwig, U. A. et al. Arbuscular mycorrhiza infection enhances the growth response of *Lolium perenne* to elevated atmospheric pCO_2. *J. Exp. Bot.* **371**, 1207-1213 (2002).

35 Hu, S., Wu, J., Burkey, K. O. & Firestone, M. K. Plant and microbial N acquisition under elevated atmospheric CO_2 in two mesocosm experiments with annual grasses. *Glob. Chang. Biol.* **11**, 213-223 (2005).

36 Ineichen, K., Wiemken, V. & Wiemken, A. Shoots, roots and ectomycorrhiza formation of pine seedlings at elevated atmospheric carbon dioxide. *Plant Cell Environ.* **18**, 703-707 (1995).

37 Izumi, H., Elfstrand, M. & Fransson, P. *Suillus* mycelia under elevated atmospheric CO_2 support increased bacterial communities and scarce *nifH* gene activity in contrast to *Hebeloma* mycelia. *Mycorrhiza* **23**, 155-165 (2013).

38 Jifon, J. L., Graham, J. H., Drouillard, D. L. & Syvertsen, J. P. Growth

depression of mycorrhizal *Citrus* seedlings grown at high phosphorus supply is mitigated by elevated CO_2. *New Phytol.* **153**, 133-142 (2002).

39 Jongen, M., Fay, P. & Jones, M. B. Effects of elevated carbon dioxide and arbuscular mycorrhizal infection on *Trifolium repens*. *New Phytol.* **132**, 413-423 (1996).

40 Kasurinen, A., Helmisaari, H. & Holopainen, T. The influence of elevated CO_2 and O_3 on fine roots and mycorrhizas of naturally growing young Scots pine trees during three exposure years. *Glob. Chang. Biol.* **5**, 771-780 (1999).

41 Klironomos, J. N. et al. Abrupt rise in atmospheric CO_2 overestimates community response in a model plant-soil system. *Nature* **433**, 621-624 (2005).

42 Klironomos, J. N., Rillig, M. C. & Allen, M. F. Below-ground microbial and microfaunal responses to *Artemisia tridentata* grown under elevated atmospheric CO_2. *Funct. Ecol.* **10**, 527-534 (1996).

43 Klironomos, J. N., Rillig, M. C., Allen, M. F., Zak, D. R., Kubiske, M. & Pregitzer, K. S. Soil fungal-arthropod responses to *Populus tremuloides* grown under enriched atmospheric CO_2 under field conditions. *Glob. Chang. Biol.* **3**, 473-478 (1997).

44 Klironomos, J. N., Ursic, M., Rillig, M. & Allen, M. F. Interspecific differences in the response of arbuscular mycorrhizal fungi to *Artemisia tridentata* grown under elevated atmospheric CO_2. *New Phytol.* **138**, 599-605 (1998).

45 Kogawara, S., Norisada, M., Tange, T., Yagi, H. & Kojima, K. Elevated atmospheric CO_2 concentration alters the effect of phosphate supply on growth of Japanese red pine (*Pinus densiflora*) seedlings. *Tree Physiol.* **26**, 25-33 (2006).

46 Kytöviita, M., Pelloux, J., Fontaine, V., Botton, B. & Dizengremel, P. Elevated

CO_2 does not ameliorate effects of ozone on carbon allocation in *Pinus halepensis* and *Betula pendula* in symbiosis with *Paxillus involutus*. *Physiol. Plant.* **106**, 370-377 (1999).

47 Kytöviita, M., Thiec, D. L. & Dizengremel, P. Elevated CO_2 and ozone reduce nitrogen acquisition by *Pinus halepensis* from its mycorrhizal symbiont. *Physiol. Plant.* **111**, 305-312 (2001).

48 Langley, J. A., Dijkstra, P., Drake, B. G. & Hungate, B. A. Ectomycorrhizal Colonization, Biomass, and Production in a Regenerating Scrub Oak Forest in Response to Elevated CO_2. *Ecosystems* **6**, 424-430 (2003).

49 Lewis, J. D., Thomas, R. B. & Strain, B. R. Effect of elevated CO_2 on mycorrhizal colonization of loblolly pine (*Pinus taeda* L.) seedlings. *Plant Soil* **165**, 81-88 (1994).

50 Loewe, A., Einig, W., Shi, L., Dizengremel, P. & Hampp, R. Mycorrhiza formation and elevated CO_2 both increase the capacity for sucrose synthesis in source leaves of spruce and aspen. *New Phytol.* **145**, 565-574 (2000).

51 Louche-Tessandier, D., Samson, G., HERNÁNDEZ-SEBASTI, C., Chagvardieff, P. & Desjardins, Y. Importance of light and CO_2 on the effects of endomycorrhizal colonization on growth and photosynthesis of potato plantlets (*Solanum tuberosum*) in an *in vitro* tripartite system. *New Phytol.* **142**, 539-550 (1999).

52 Lukac, M., Calfapietra, C. & Godbold, D. Production, turnover and mycorrhizal colonization of root systems of three *Populus* species grown under elevated CO_2 (POPFACE). *Glob. Chang. Biol.* **9**, 838-848 (2003).

53 Lussenhop, J., Treonis, A., Curtis, P. S., Teeri, J. A. & Vogel, C. S. Response of soil biota to elevated atmospheric CO_2 in poplar model systems. *Oecologia* **113**,

247-251 (1998).

54 Maček, I. et al. Impacts of long-term elevated atmospheric CO_2 concentrations on communities of arbuscular mycorrhizal fungi. *Mol. Ecol.* **28**, 2445-3458 (2019).

55 Markkola, A. M., Ohtonen, A., Ahonen-Jonnarth, U. & Ohtonen, R. Scots pine responses to CO_2 enrichment-I. Ectomycorrhizal fungi and soil fauna. *Environ. Pollut.* **94**, 309-316 (1996).

56 Nowak, J. & Nowak, J. S. CO_2 enrichment and mycorrhizal effects on cutting growth and some physiological traits of cuttings during rooting. *Ogrodnictwo* **12**, 67-75 (2013).

57 Olesniewicz, K. S. & Thomas, R. B. Effects of mycorrhizal colonization on biomass production and nitrogen fixation of black locust (*Robinia pseudoacacia*) seedlings grown under elevated atmospheric carbon dioxide. *New Phytol.* **142**, 133-140 (1999).

58 O'Neill, E. G., Luxmoore, R. J. & Norby, R. J. Increases in mycorrhizal colonization and seedling growth in *Pinus echinata* and *Quercus alba* in an enriched CO_2 atmosphere. *Can. J. For. Res.* **17**, 878-883 (1987).

59 Panneerselvam, P. et al. Influence of elevated CO_2 on arbuscular mycorrhizal fungal community elucidated using Illumina MiSeq platform in sub-humid tropical paddy soil. *Appl. Soil Ecol.* **145**, 103344 (2020).

60 Panneerselvam, P., Sahoo, S., Senapati, A., Kumar, U., Mitra, D. & Parameswaran, C. Understanding interaction effect of arbuscular mycorrhizal fungi in rice under elevated carbon dioxide conditions. *J. Basic Microbiol.* **59**, 1217-1228 (2019).

61 Plett, J. M. et al. The effect of elevated carbon dioxide on the interaction

between *Eucalyptus grandis* and diverse isolates of *Pisolithus* sp. is associated with a complex shift in the root transcriptome. *New Phytol.* **206**, 1423-1436 (2014).

62 Rillig, M. C. & Field, C. B. Arbuscular mycorrhizae respond to plants exposed to elevated atmospheric CO_2 as a function of soil depth. *Plant Soil* **254**, 383-391 (2003).

63 Rillig, M. C., Allen, M. F., Klironomos, J. N. & Field, C. B. Arbuscular mycorrhizal percent root infection and infection intensity of *Bromus hordeaceus* grown in elevated atmospheric CO_2. *Mycologia* **90**, 199-205 (1998).

64 Rillig, M. C., Allen, M. F., Klironomos, J. N., Chiariello, N. R. & Field, C. B. Plant species-specific changes in root-inhabiting fungi in a California annual grassland: responses to elevated CO_2 and nutrients. *Oecologia* **113**, 252-259 (1998).

65 Rillig, M. C., Field, C. B. & Allen, M. F. Fungal root colonization responses in natural grasslands after long-term exposure to elevated atmospheric CO_2. *Glob. Chang. Biol.* 5, 577-585 (1999).

66 Rillig, M. C., Field, C. B. & Allen, M. F. Soil biota responses to long-term atmospheric CO_2 enrichment in two California annual grasslands. *Oecologia* **119**, 572-577 (1999).

67 Rønn, R., Gavito, M., Larsen, J., Jakobsen, I., Frederiksen, H. & Christensen, S. Response of free-living soil protozoa and microorganisms to elevated atmospheric CO_2 and presence of mycorrhiza. *Soil Biol. Biochem.* **34**, 923-932 (2002).

68 Rouhier, H. & Read, D. J. Plant and fungal responses to elevated atmospheric carbon dioxide in mycorrhizal seedlings of *Pinus sylvestris*. *Environ. Exp. Bot.*

40, 237-246 (1998).

69 Rouhier, H. & Read, D. J. Plant and fungal responses to elevated atmospheric CO_2 in mycorrhizal seedlings of *Betula pendula*. *Environ. Exp. Bot.* **42**, 231-241 (1999).

70 Rouhier, H. & Read, D. J. The role of mycorrhiza in determining the response of *Plantago lanceolata* to CO_2 enrichment. *New Phytol.* **139**, 367-373 (1998).

71 Rúa, M. A., Umbanhowar, J., Hu, S., Burkey, K. O. & Mitchell, C. E. Elevated CO_2 spurs reciprocal positive effects between a plant virus and an arbuscular mycorrhizal fungus. *New Phytol.* **199**, 541-549 (2013).

72 Runion, G. B., Curl, E. A., Rogers, H. H., Backman, P. A., Rodriguez-Kábana, R. & Helms, B. E. Effects of free-air CO_2 enrichment on microbial populations in the rhizosphere and phyllosphere of cotton. *Agric. For. Meteorol.* **70**, 117-130 (1994).

73 Sanders, I. R., Streitwolf-Engel, R., van der Heijden, M. G. A., Boller, T. & Wiemken, A. Increased allocation to external hyphae of arbuscular mycorrhizal fungi under CO_2 enrichment. *Oecologia* **117**, 496-503 (1998).

74 Shinano, T., Yamamoto, T., Tawaraya, K., Tadokoro, M., Koike, T. & Osaki, M. Effects of elevated atmospheric CO_2 concentration on the nutrient uptake characteristics of Japanese larch (*Larix kaempferi*). *Tree Physiol.* **27**, 97-104 (2007).

75 Staddon, P. L., Fitter, A. H. & Graves, J. D. Effect of elevated atmospheric CO_2 on mycorrhizal colonization, external mycorrhizal hyphal production and phosphorus inflow in *Plantago lanceolata* and *Trifolium repens* in association with the arbuscular mycorrhizal fungus *Glomus mosseae*. *Glob. Chang. Biol.* **5**, 347-358 (1999).

76 Staddon, P. L., Graves, J. D. & Fitter, A. H. Effect of enhanced atmospheric CO_2 on mycorrhizal colonization by *Glomus mosseae* in *Plantago lanceolata* and *Trifolium repens*. *New Phytol.* **139**, 571-580 (1998).

77 Staddon, P. L., Jakobsen, I. & Blum, H. Nitrogen input mediates the effect of free-air CO_2 enrichment on mycorrhizal fungal abundance. *Glob. Chang. Biol.* **10**, 1678-1688 (2004).

78 Staddon, P. L., Reinsch, S., Olsson, P. L., Ambus, P., Lüscher, A. & Jakobsen, I. A decade of free-air CO_2 enrichment increased the carbon throughput in a grass-clover ecosystem but did not drastically change carbon allocation patterns. *Funct. Ecol.* **28**, 538-545 (2014).

79 Staddon, P., Gregersen, R. & Jakobsen, I. The response of two *Glomus* mycorrhizal fungi and a fine endophyte to elevated atmospheric CO_2, soil warming and drought. *Glob. Chang. Biol.* **10**, 1909-1921 (2004).

80 Syvertsen, J. P. & Graham, J. H. Phosphorus supply and arbuscular mycorrhizas increase growth and net gas exchange responses of two *Citrus* spp. grown at elevated [CO_2]. *Plant Soil* **208**, 209-219 (1999).

81 Tang, J., Chen, J. & Chen, X. Response of 12 weedy species to elevated CO_2 in low-phosphorus-availability soil. *Ecol. Res.* **21**, 664-670 (2006).

82 Tang, J., Xu, L., Chen, X. & Hu, S. Interaction between C_4 barnyard grass and C_3 upland rice under elevated CO_2: Impact of mycorrhizae. *Acta Oecol.* **35**, 227-235 (2009).

83 Taylor, B. N., Strand, A. E., Cooper, E. R., Beidler, K. V., Schonholz, M. & Pritchard, S. G. Root length, biomass, tissue chemistry and mycorrhizal colonization following 14 years of CO_2 enrichment and 6 years of N fertilization in a warm temperate forest. *Tree Physiol.* **34**, 955-965 (2014).

84 Thirkell, T. J., Pastok, D. & Field, K. J. Carbon for nutrient exchange between arbuscular mycorrhizal fungi and wheat varies according to cultivar and changes in atmospheric carbon dioxide concentration. *Glob. Chang. Biol.* **26**, 1725-1738 (2020).

85 Treseder, K. K., Egerton-Warburton, L. M., Allen, M. F., Cheng, Y. & Oechel, W. C. Alteration of soil carbon pools and communities of mycorrhizal fungi in chaparral exposed to elevated carbon dioxide. *Ecosystems* **6**, 786-796 (2003).

86 Turnau, K. et al. Carbon dioxide concentration and nitrogen input affect the C and N storage pools in *Amanita muscaria-Picea abies* mycorrhizae. *Tree Physiol.* **21**, 93-99 (2001).

87 Walker, R. F., Geisinger, D. R., Johnson, D. W. & Ball, J. T. Atmospheric CO_2 enrichment and soil N fertility effects on juvenile ponderosa pine: growth, ectomycorrhizal development, and xylem water potential. *For. Ecol. Manage.* **102**, 33-44 (1998).

88 Walker, R. F., Geisinger, D. R., Johnson, D. W. & Ball, J. T. Elevated atmospheric CO_2 and soil N fertility effects on growth, mycorrhizal colonization, and xylem water potential of juvenile ponderosa pine in a field soil. *Plant Soil* **195**, 25-36 (1997).

89 Walker, R. F., Geisinger, D. R., Johnson, D. W. & Ball, J. T. Enriched atmospheric CO_2 and soil P effects on growth and ectomycorrhizal colonization of juvenile ponderosa pine. *For. Ecol. Manage.* **78**, 207-215 (1995).

90 Walker, R. F., Johnson, D. W., Geisinger, D. R. & Ball, J. T. Growth and ectomycorrhizal colonization of ponderosa pine seedlings supplied different levels of atmospheric CO_2 and soil N and P. *For. Ecol. Manage.* **109**, 9-20 (1998).

91 Wang, X., Li, S., Bai, K. & Kuang, T. Influences of double aerial CO_2 concentration on plant root surface area and viability and infection intensity of vesicular-arbuscular mycorrhizal fungi. *Chin. Sci. Bull.* **44**, 63-64 (1999).

92 Wang, X. et al. Ectomycorrhizal colonization and growth of the hybrid larch F_1 under elevated CO_2 and O_3. *Environ. Pollut.* **197**, 116-126 (2015).

93 Watts-Williams, S. J., Andrew Smith, F. & Jakobsen, I. Soil phosphorus availability is a driver of the responses of maize (*Zea mays*) to elevated CO_2 concentration and arbuscular mycorrhizal colonisation. *Symbiosis* **77**, 73-82 (2019).

94 Weigt, R. B., Raidl, S., Verma, R., Rodenkirchen, H., GöTtlein, A. & Agerer, R. Effects of twice-ambient carbon dioxide and nitrogen amendment on biomass, nutrient contents and carbon costs of norway spruce seedlings as influenced by mycorrhization with *Piloderma croceum* and *Tomentellopsis submollis*. *Mycorrhiza* **21**, 375-391 (2011).

95 Werner, G. D. A., Zhou, Y., Pieterse, C. M. J. & Kiers, E. T. Tracking plant preference for higher-quality mycorrhizal symbionts under varying CO_2 conditions over multiple generations. *Ecol. Evol.* **8**, 78-87 (2018).

96 Wiemken, V., Ineichen, K. & Boller, T. Development of ectomycorrhizas in model beech-spruce ecosystems on siliceous and calcareous soil: a 4-year experiment with atmospheric CO_2 enrichment and nitrogen fertilization. *Plant Soil* **234**, 99-108 (2001).

97 Zhou, Y. et al. A novel CO_2-responsive systemic signaling pathway controlling plant mycorrhizal symbiosis. *New Phytol.* **224**, 106-116 (2019).

98 Zhu, X., Song, F., Liu, S. & Liu, F. Arbuscular mycorrhiza improve growth, nitrogen uptake, and nitrogen use efficiency in wheat grown under elevated CO_2.

Mycorrhiza **26**, 133-140 (2015).

99 Zhu, X., Song, F., Liu, S. & Liu, F. Role of arbuscular mycorrhiza in alleviating salinity stress in wheat (*Triticum aestivum* L.) grown under ambient and elevated CO_2. *J. Agron. Crop Sci.* **202**, 486-496 (2016).

100 Zhu, X., Song, F., Liu, S., Liu, F. & Li, X. Arbuscular mycorrhiza enhances nutrient accumulation in wheat exposed to elevated CO_2 and soil salinity. *J. Plant Nutr. Soil Sci.* **181**, 836-846 (2018).

Supplementary Methods: References used to analyze the effects of experimental warming on mycorrhiza symbiosis

1 Clemmensen, K. E., Michelsen, A., Jonasson, S. & Shaver, G. R. Increased ectomycorrhizal fungal abundance after long-term fertilization and warming of two arctic tundra ecosystems. *New Phytol.* **171**, 391-404 (2006).

2 Deslippe, J. R., Hartmann, M., Mohn, W. W. & Simard, S. W. Long-term experimental manipulation of climate alters the ectomycorrhizal community of Betula nana in Arctic tundra. *Glob. Chang. Biol.* **17**, 1625-1636 (2011).

3 Fujimura, K. E., Egger, K. N. & Henry, G. HR. The effect of experimental warming on the root-associated fungal community of *Salix arctica*. *ISME J.* **2**, 105-114 (2008).

4 Gao, C. et al. Increased precipitation, rather than warming, exerts a strong influence on arbuscular mycorrhizal fungal community in a semiarid steppe ecosystem. *Botany* **94**, 459-469 (2016).

5 Gavito, M. E., Schweiger, P. & Jakobsen, I. P uptake by arbuscular mycorrhizal hyphae: effect of soil temperature and atmospheric CO_2 enrichment. *Glob. Chang. Biol.* **9**, 106-116 (2003).

6 Gpey, W. E. Influence of temperature on colonization of spring barleys by vesicular arbuscular mycorrhizal fungi. *Plant Soil* **137**, 181-190 (1991).

7 Heinemeyer, A. & Fitter, A. H. Impact of temperature on the arbuscular mycorrhizal (AM) symbiosis: growth responses of the host plant and its AM fungal partner. *J. Exp. Bot.* **55**, 525-534 (2004).

8 Heinemeyer, A., Ridgway, K. P., Edwards, E. J., Benham, D. G., Young, J. P. & Fitter, A. H. Impact of soil warming and shading on colonization and community structure of arbuscular mycorrhizal fungi in roots of a native

grassland community. *Glob. Chang. Biol.* **10**, 52-64 (2003).

9 Hu, Y. et al. Arbuscular mycorrhizal symbiosis can mitigate the negative effects of night warming on physiological traits of *Medicago truncatula* L. *Mycorrhiza* **25**, 131-142 (2015).

10 Kim, Y. C. et al. Arbuscular mycorrhizal fungal community response to warming and nitrogen addition in a semiarid steppe ecosystem. *Mycorrhiza* **25**, 267-276 (2015).

11 Kim, Y. et al. Different responses of arbuscular mycorrhizal fungal community to day-time and night-time warming in a semiarid steppe. *Chin. Sci. Bull.* **59**, 5080-5089 (2014).

12 Mei, L., Yang, X., Zhang, S., Zhang, T. & Guo, J. Arbuscular mycorrhizal fungi alleviate phosphorus limitation by reducing plant N:P ratios under warming and nitrogen addition in a temperate meadow ecosystem. *Sci. Total Environ.* **686**, 1129-1139 (2019).

13 Monz, C. A., Hunt, H. W., Reeves, F. B. & Elliott, E. T. The response of mycorrhizal colonization to elevated CO_2 and climate change in *Pascopyrum smithii* and *Bouteloua gracilis*. *Plant Soil* **165**, 75-80 (1994).

14 Olsrud, M., Melillo, J. M., Christensen, T. R., Michelsen, A., Wallander, H. & Olsson, P. A. Response of ericoid mycorrhizal colonization and functioning to global change factors. *New Phytol.* **162**, 459-469 (2004).

15 Qiao, M. et al. Experimental warming effects on root nitrogen absorption and mycorrhizal infection in a subalpine coniferous forest. *Scand. J. For. Res.* **31**, 347-354 (2016).

16 Rillig, M. C., Wright, S. F., Shaw, M. R. & Field, C. B. Artificial climate warming positively affects arbuscular mycorrhizae but decreases soil aggregate

water stability in an annual grassland. *Oikos* **97**, 52-58 (2002).

17 Staddon, P. L., Gregersen, R. & Jakobsen, I. The response of two *Glomus* mycorrhizal fungi and a fine endophyte to elevated atmospheric CO_2, soil warming and drought. *Glob. Chang. Biol.* **10**, 1909-1921 (2004).

18 Staddon, P. L., Thompson, K., Jakobsen, I., Grime, G. P., Askew, A. & Fitter, A. H. Mycorrhizal fungal abundance s affected by long-term climatic manipulations in the field. *Glob. Chang. Biol.* **9**, 186-194 (2003).

19 Sun, X., Su, Y., Zhang, Y., Wu, M., Zhang, Z. & Pei, K. Diversity of arbuscular mycorrhizal fungal spore communities and its relations to plants under increased temperature and precipitation in a natural grassland. *Chin. Sci. Bull.* **58**, 4109-4119 (2013).

20 Wilson, H., Johnson, B. R., Bohannan, B., Pfeifer-Meister, L., Mueller, R. & Bridgham, S. D. Experimental warming decreases arbuscular mycorrhizal fungal colonization in prairie plants along a Mediterranean climate gradient. *Peerj* **4**, e2083 (2016).

21 Yang, W. et al. The arbuscular mycorrhizal fungal community response to warming and grazing differs between soil and roots on the Qinghai-Tibetan Plateau. *PLoS ONE* **8**, e76447 (2013).

22 Zhang, T., Yang, X., Guo, R. & Guo, J. Response of AM fungi spore population to elevated temperature and nitrogen addition and their influence on the plant community composition and productivity. *Sci. Rep.* **6**, 24749 (2016).

Supplementary Figures

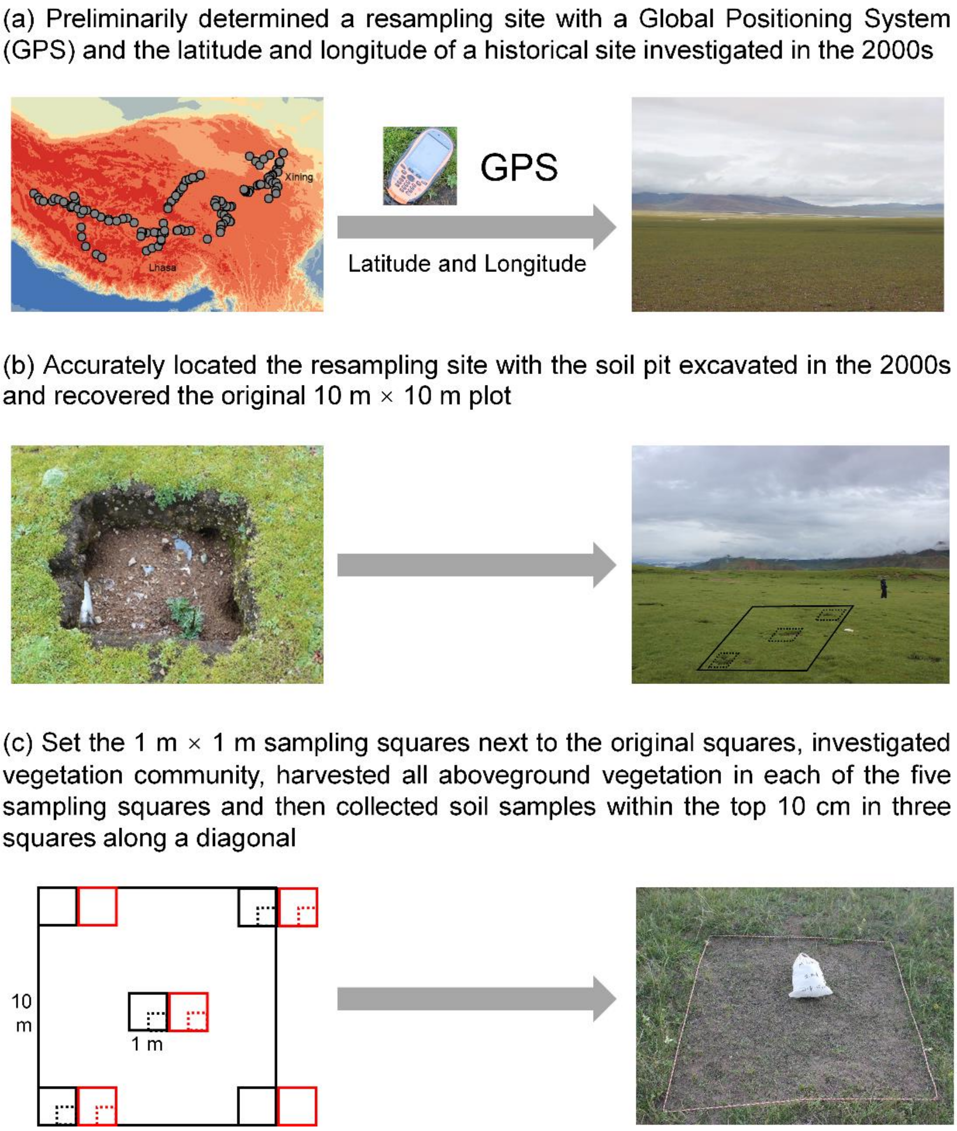

Supplementary Fig. 1. The schematic diagram for the Tibetan resampling campaign.

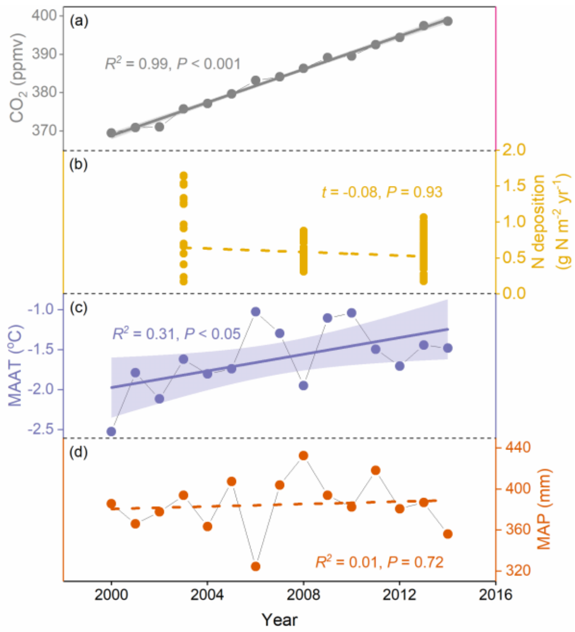

Supplementary Fig. 2. Environmental changes on the Tibetan Plateau. Panels (a-d) represent changes in atmospheric carbon dioxide (CO_2) concentration, nitrogen (N) deposition, mean annual air temperature (MAAT) and mean annual precipitation (MAP) over 2000s~2010s, respectively. Data for atmospheric CO_2 concentration, N deposition and climate (MAAT and MAP) are derived from Chinese Research Network or Special Environment and Disaster (http://www.crensed.ac.cn), Science Data Bank (http://www.csdata.org/p/199/) and China Meteorological Administration (http://data.cma.cn/), respectively. The changes in CO_2 concentration and climate (MAAT and MAP) were examined with Ordinary Least Square regressions, and the change in N deposition was detected with linear mixed-effects model, in which the fixed effect was year and the random effect was site. The solid fitted lines indicate significant environmental changes, while the dashed fitted lines denote insignificant dynamics. The shade accompanying with the solid fitted line represents 95% confidential interval.

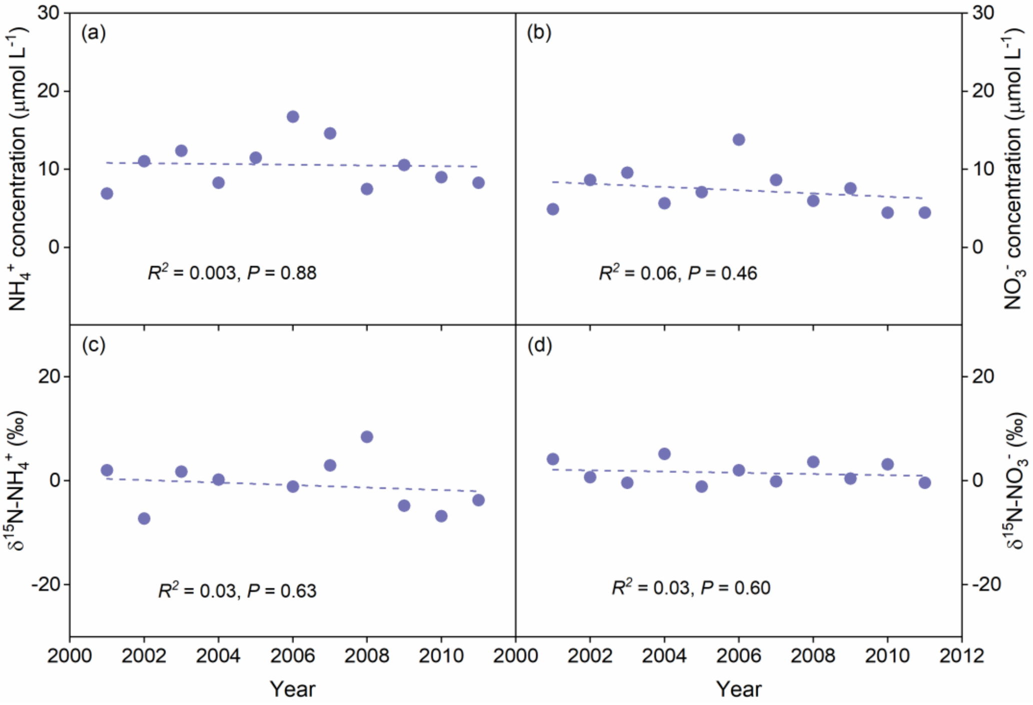

Supplementary Fig. 3. Changes in atmospheric N deposition and its $\delta^{15}N$. Panels (a-d) represent ammonium (NH_4^+) concentration, nitrate (NO_3^-) concentration, $\delta^{15}N$-NH_4^+, and $\delta^{15}N$-NO_3^- derived from a representative ice core on the Tibetan Plateau during the period from 2001 to 2011. The N concentration and $\delta^{15}N$ in ice cores have widely used to reflect the dynamics of atmospheric nitrogen (N) deposition and its isotopic characteristics[8,9,10]. In our case, to characterize changes in $\delta^{15}N$ values of atmospheric N deposition, we obtained isotopic observations based on an ice core from our co-author (Dr. Yunting Fang)[11]. The changes in N concentration and $\delta^{15}N$ were examined with Ordinary Least Square regressions, and the dashed fitted lines denote insignificant changes.

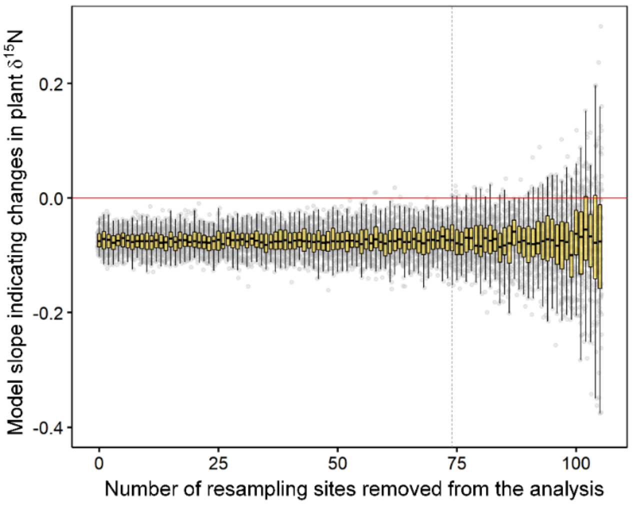

Supplementary Fig. 4. Changes in plant $\delta^{15}N$ dynamics with the resampling size. Plant $\delta^{15}N$ dynamics was detected by the bootstrapped estimate of model slope after a specific number of resampling sites were randomly removed, of which the model refers to the linear mixed-effects model used to statistically analyze changes in plant $\delta^{15}N$ on the Tibetan Plateau over the period from the 2000s to the 2010s. This analysis was used to estimate the threshold that made the findings of changes in plant $\delta^{15}N$ lose statistical significance once we randomly removed sites from the original dataset. In the analyses, the bootstrap was iterated for 10,000 times, in each of which the resampling sites were removed randomly and the slope of the linear mixed-effects model used to test changes in plant $\delta^{15}N$ was estimated based on the remaining sites[12]. The estimated slopes were then used to calculate interquartile range for each number of resampling sites that were removed from the dataset[12]. The average slope was considered non-significant if the interquartile range of the slope overlapped with the horizontal zero line[12]. The dashed line represents the first interquartile range which overlapped with the horizontal zero line. Each point in the diagram represents a bootstrapped estimate of model slope, and each box-plot presents the interquartile range and median slope for each number of resampling sites removed from the analysis.

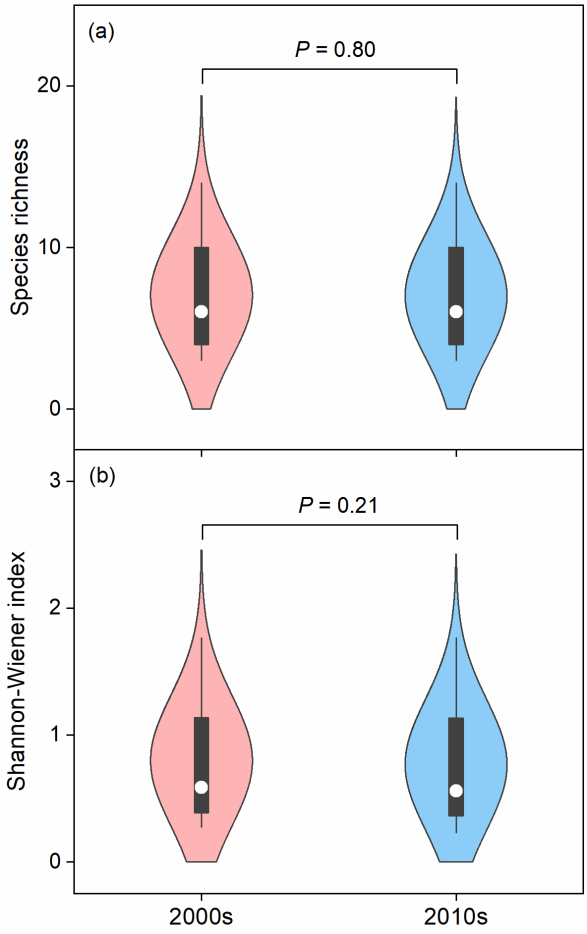

Supplementary Fig. 5. Changes in vegetation composition at the community level. Panels (a-b) represent changes in species richness and Shannon-Wiener index across the resampling sites on the Tibetan Plateau during the period from the 2000s to the 2010s, respectively. Species richness refers to species number per quadrat, and Shannon-Wiener index (H) was calculated as: $H = -\sum P_i \ln P_i$, where P_i is the relative cover of species *i*[1]. The change in vegetation composition was examined with linear mixed-effects model, in which the fixed effect was sampling years and the random effects were the investigated sites as well as replicates within each site. Numbers near each box diagram denote the percentile of 95%, 75%, 50%, 25% and 5%, respectively.

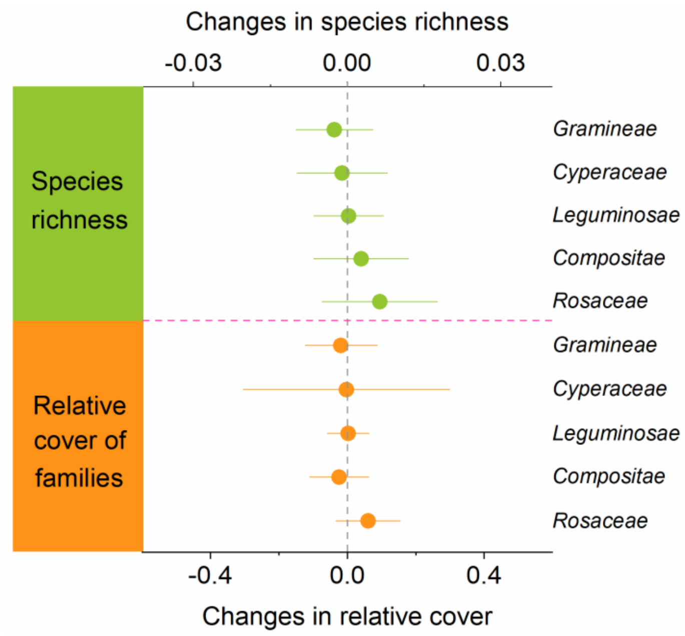

Supplementary Fig. 6. Changes in vegetation composition at the family level. During this analysis, changes in species richness and the relative cover of five families that can be heavily colonized by mycorrhizae were detected on the Tibetan Plateau over the period from the 2000s to the 2010s. The species richness here refers to species number per quadrat for each of the five families, and the relative cover was calculated as the sum of the relative cover of each species within a specific family[1]. To conduct the family-level analysis, we selected five families that are heavily colonized by mycorrhizae consisted of *Gramineae*, *Cyperaceae*, *Leguminosae*, *Compositae* and *Rosaceae*[3]. The changes in species richness and the relative cover were examined with linear mixed-effects models, in which the fixed effect was sampling years and the random effects were the investigated sites as well as replicates within each site. Points in the plot indicate the estimated model slopes, and error bars denote 95% confidence intervals. The change in species richness or the relative cover is considered non-significant if the 95% confidence interval overlaps with the vertical zero line.

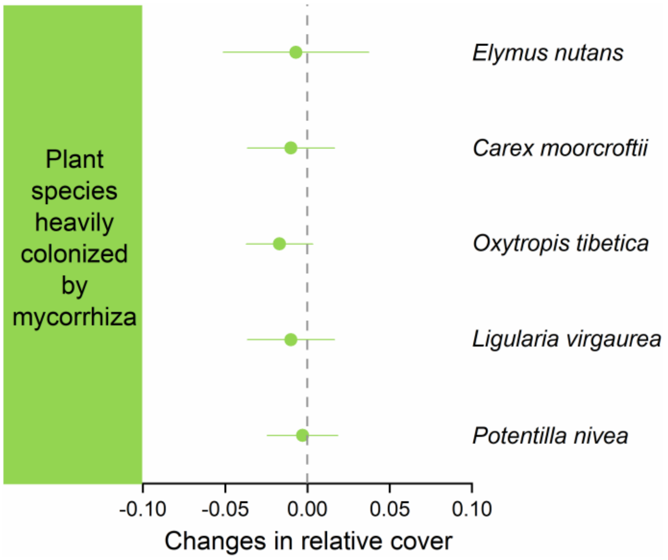

Supplementary Fig. 7. Changes in vegetation composition at the species level. During this analysis, changes in the relative cover of host plant species that can be heavily colonized by mycorrhizae were detected on the Tibetan Plateau over the period from the 2000s to the 2010s. The host plant species *Elymus nutans*, *Carex moorcroftii*, *Oxytropis tibetica*, *Ligularia virgaurea* and *Potentilla nivea* belong to the plant family *Gramineae*, *Cyperaceae*, *Leguminosae*, *Compositae* and *Rosaceae*, respectively[3]. The change in species richness and the relative cover was examined with linear mixed-effects models, in which the fixed effect was sampling years and the random effects were the investigated sites as well as replicates within each site. Points in the plot indicate the estimates of model slopes, and the error bars denote 95% confidence intervals. The change in the relative cover is considered non-significant if the 95% confidence interval overlaps with the vertical zero line.

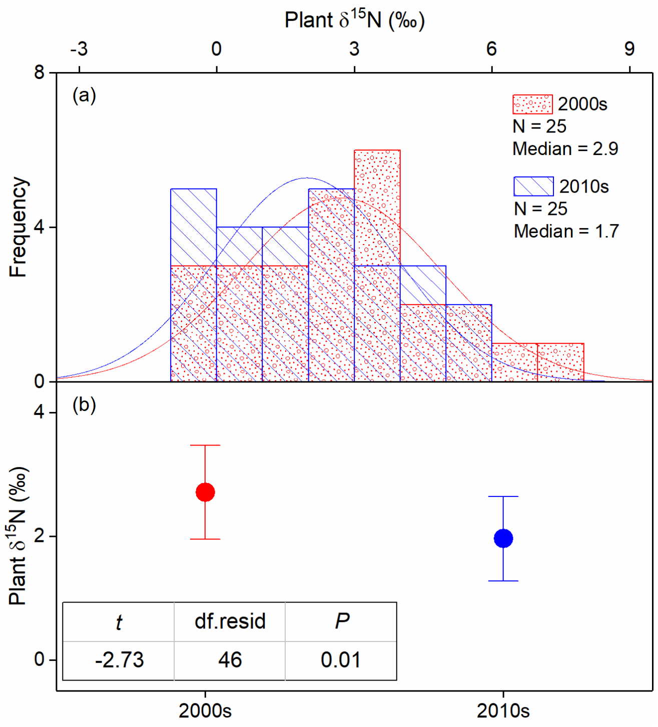

Supplementary Fig. 8. Changes in plant $\delta^{15}N$ among sites without *Leguminosae*. Panels (a-b) represent frequency distributions of plant $\delta^{15}N$ among those resampling sites without the *Leguminosae* during the two sampling periods, and changes in the corresponding plant $\delta^{15}N$ over the detection period, respectively. The change in plant $\delta^{15}N$ was examined with linear mixed-effects model, in which the fixed effect was year and the random effect was sampling site. Points in panel (b) denote mean values and error bars represent 95% confidence intervals. N, the number of sites used for analyzing plant $\delta^{15}N$; Median, median value of plant $\delta^{15}N$; df.resid, residual degrees of freedom.

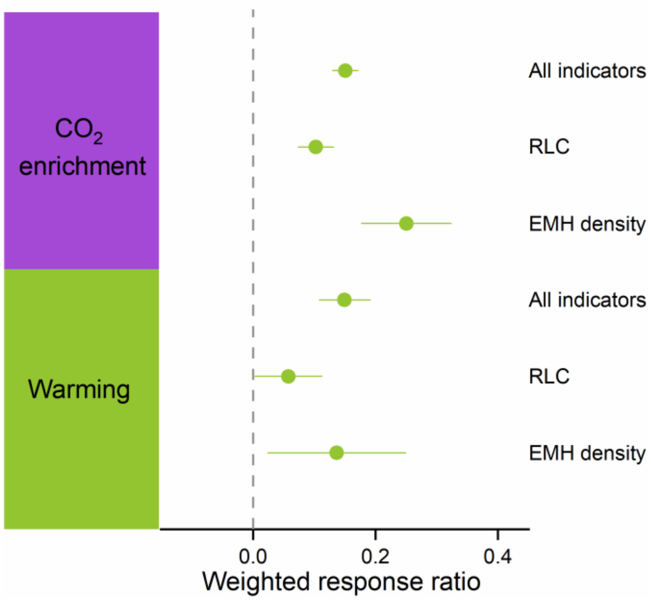

Supplementary Fig. 9. Effects of environmental change on mycorrhizal symbiosis. During this analysis, the weighted response ratios reflecting CO_2 enrichment or warming effects on mycorrhizal symbiosis were estimated based on meta-analyses of published literature (Supplementary Note 2; Supplementary Methods). The all indicators mean all the collected variables associated with mycorrhizal symbiosis. RLC represents the percentage of root length colonized. EMH denotes extraradical mycorrhizal hyphal. Points in the plot denote ln-transformed weighted response ratios, and error bars represent 95% confidence intervals. The vertical dashed line is drawn at the weighted response ratio = 0. The CO_2 enrichment or warming effect on mycorrhizal symbiosis was considered non-significant if the 95% confidence interval overlapped with the dashed vertical line.

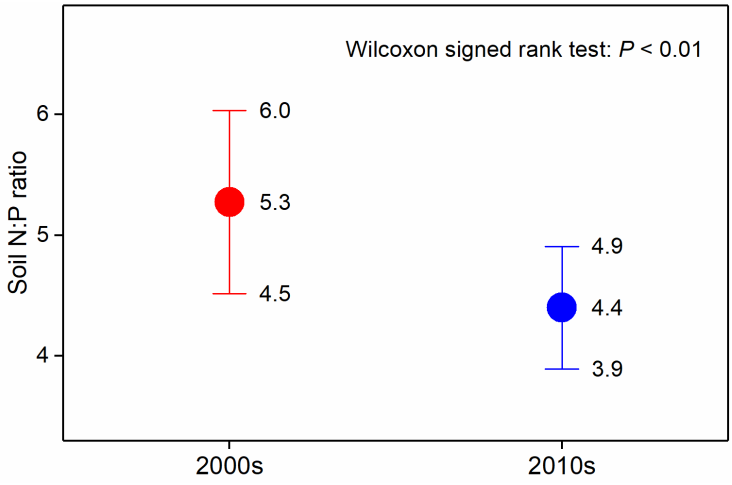

Supplementary Fig. 10. Changes in topsoil N:P ratio over 2000s~2010s. Soil N:P ratio refers to the ratio of soil nitrogen (N) content to phosphorus (P) content in the top 10 cm on the Tibetan Plateau. Soil N and P contents were measured based on samples collected across the resampling sites. The change in soil N:P ratio was examined with Wilcoxon signed rank test due to skewed distributions[13]. Points in the plot denote mean values and error bars represent 95% confidence intervals.

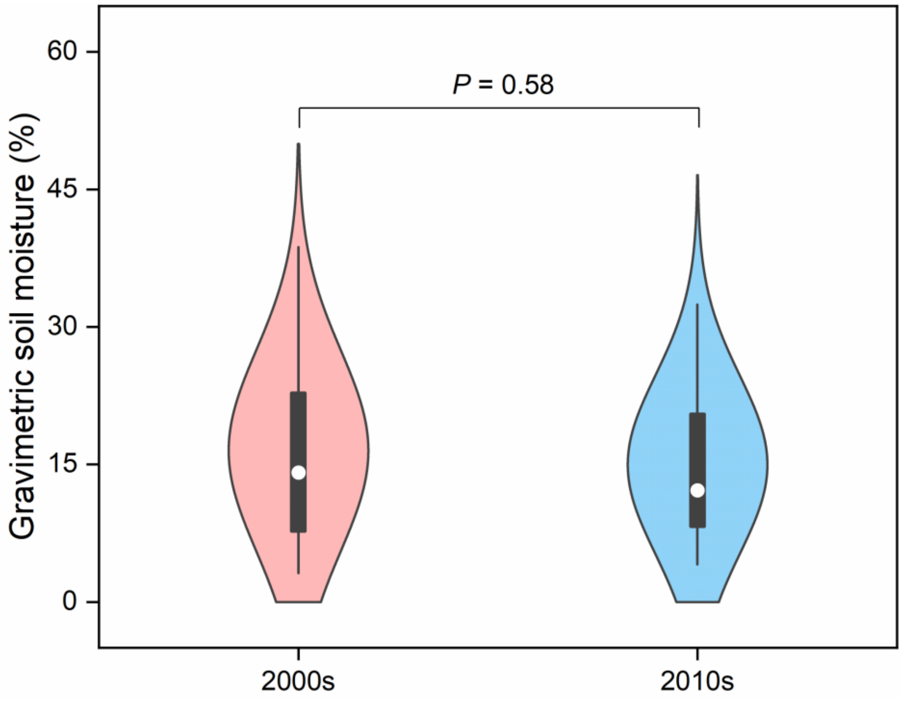

Supplementary Fig. 11. Comparison of soil water status during the two sampling periods. The soil water status refers to gravimetric soil moisture in the top 10 cm on the Tibetan Plateau. Soil moisture data were derived from the resampling investigations conducted in this study. Soil moisture dynamics was examined with linear mixed-effects model, in which the fixed effect was year and the random effect was sampling site. Numbers near each box diagram denote the percentile of 95%, 75%, 50%, 25% and 5%, respectively.

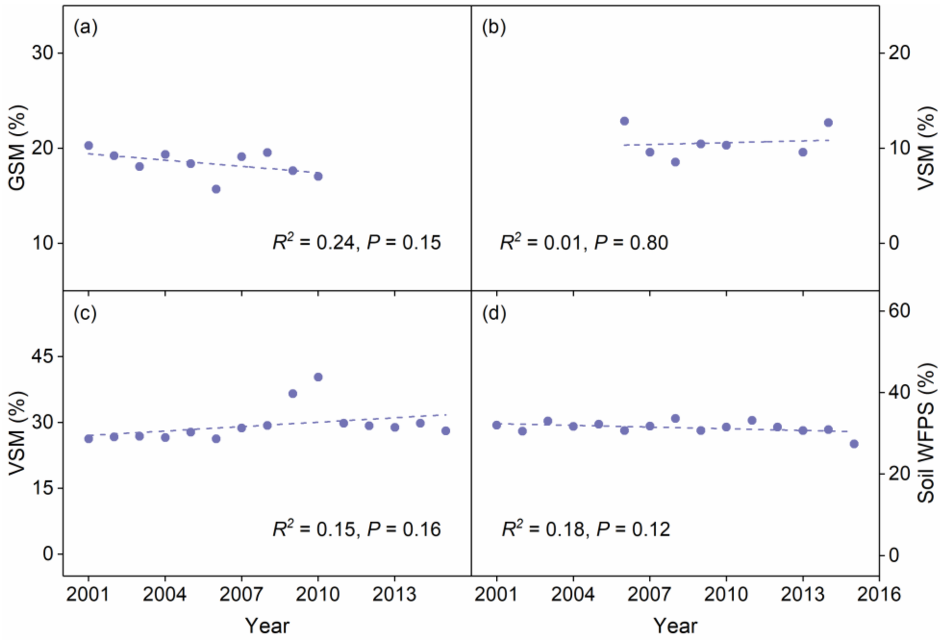

Supplementary Fig. 12. Changes in topsoil water status over 2000s~2010s. Panels (a-d) represent changes in soil water status within the top 10 cm depth derived from China Meteorological Administration (http://data.cma.cn/, a), Zhao et al. (2019)[6] (b), CFSV2 (NCEP Climate Forecast System Version 2, https://rda.ucar.edu/datasets/ds094.0/, c) and the output of DeNitrification-DeComposition (DNDC) model (d) on the Tibetan Plateau from the 2000s to the 2010s, respectively. Based on these datasets, changes in soil water status were examined with Ordinary Least Square regressions, and the dashed fitted lines in the diagram denoted insignificant trends. GSM: gravimetric soil moisture; VSM, volumetric soil moisture; WFPS, water-filled pore space.

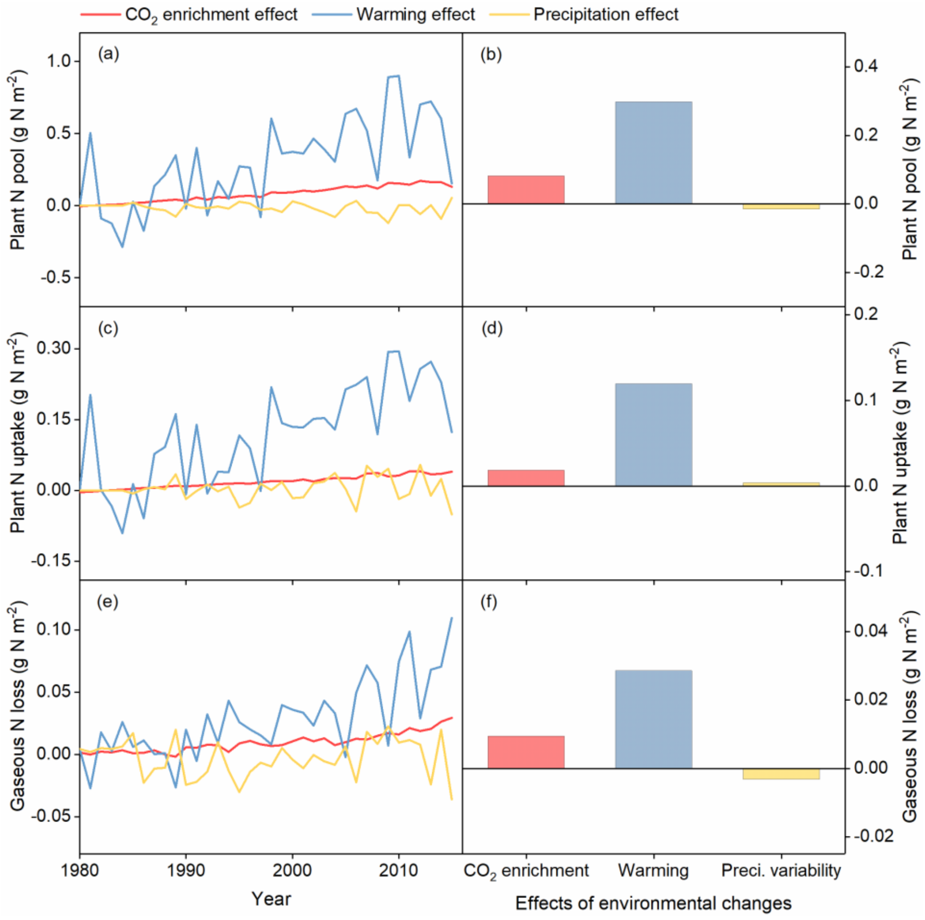

Supplementary Fig. 13. Effects of environmental change on Tibetan N cycle. Panels (a-f) represent effects of environmental changes, including enrichment of atmospheric carbon dioxide (CO_2) concentration, climate warming and precipitation variability, on plant nitrogen (N) pool (a-b), annual plant N uptake rate (c-d) and annual gaseous N loss rate (e-f) during the past decades, respectively. Effects of environmental changes on the related N pools or N cycling processes were analyzed with the simulation experiments performed by the DeNitrification-DeComposition (DNDC) model. Preci. refers to precipitation.

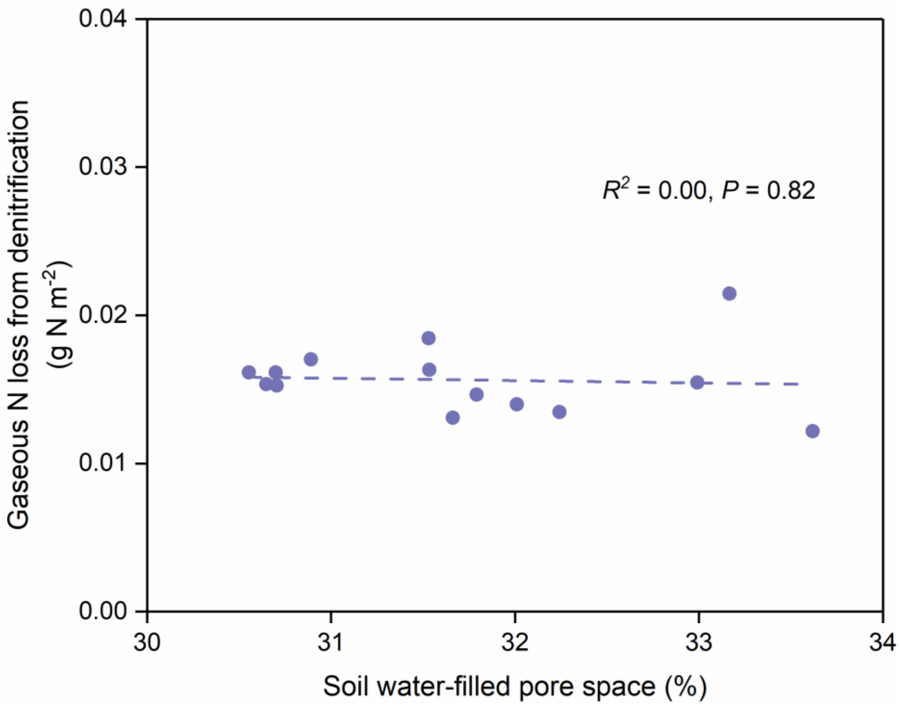

Supplementary Fig. 14. Effects of soil water status on gaseous N loss. Gaseous N losses are each year's total gaseous N loss from denitrification over the period from 2001 to 2014, and soil water status refers to the corresponding annual average soil water-filled pore space. Both gaseous N loss and soil water-filled pore space are derived from the simulation output of the DeNitrification-DeComposition (DNDC) model, and their relationship is examined with Least Ordinary Square regression. The dashed fitted line denotes an insignificant relationship.

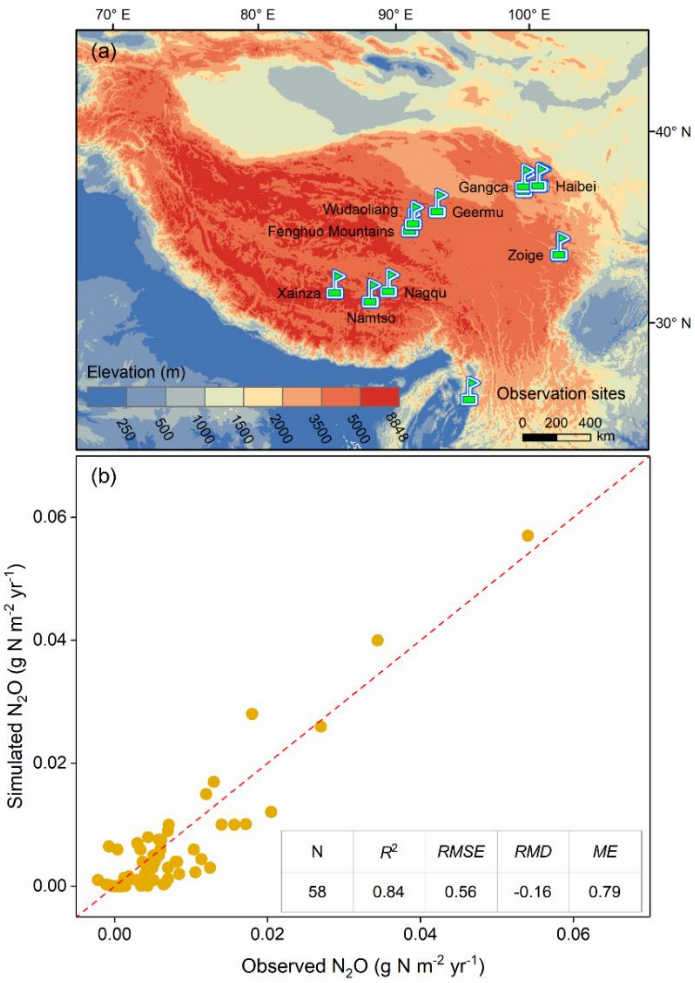

Supplementary Fig. 15. Validation of the DNDC model based on flux measurements. Panels (a-b) represent location of the observation sites used to validate the DNDC model, and performance of the validated model detected by its simulation in ecosystem nitrous oxide (N_2O) emissions, respectively. The N_2O observations were derived from publications across the study area (Supplementary Methods). The background map in panel (a) represents the elevation across the study area. The dashed line in panel (b) denotes the 1:1 line. Four indicators including R^2 (coefficient of determination), *RMSE* (root mean square error), *RMD* (relative mean deviation), and *ME* (model efficiency), were used to evaluate the model performance. N, the number of observation-simulation pairs.

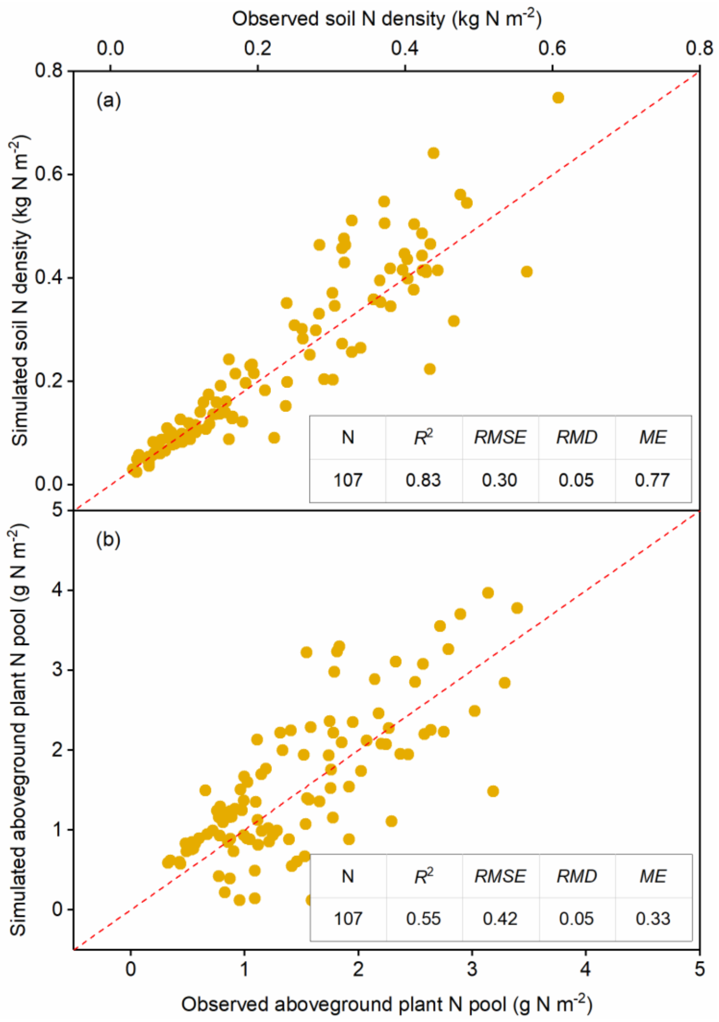

Supplementary Fig. 16. Validation of the DNDC model based on pool measurements. Panels (a-b) represent validation of the DNDC model for its simulations in soil N density within the top 10 cm and aboveground plant N pool, respectively. The observed soil N density and plant N pool were derived from the field campaign in this study. The dashed line denotes the 1:1 line. Four indicators, including R^2 (coefficient of determination), *RMSE* (root mean square error), *RMD* (relative mean deviation), and *ME* (model efficiency), were used to evaluate the model performance. N, the number of observation-simulation pairs.

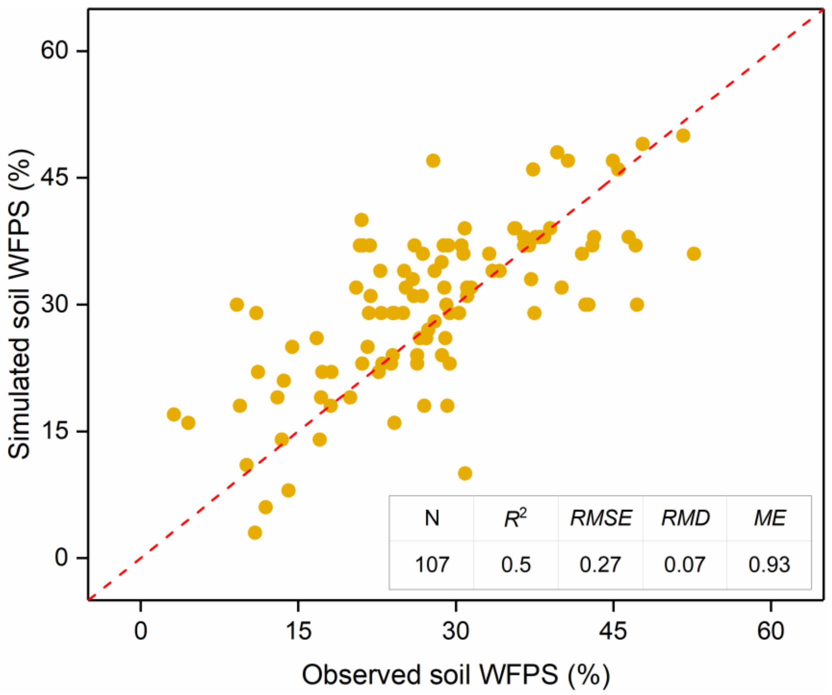

Supplementary Fig. 17. Validation of the DNDC model based on soil water status. During this analysis, the model performance was validated through its simulation in soil water-filled pore space (WFPS) across the 107 resampling sites on the Tibetan Plateau. The observed soil WFPS was calculated based on gravimetric soil moisture and bulk density[14] in the top 10 cm detected through the repeated field sampling campaign. The dashed line denotes the 1:1 line. Four indicators, including R^2 (coefficient of determination), *RMSE* (root mean square error), *RMD* (relative mean deviation), and *ME* (model efficiency), were used to evaluate the model performance. N, the number of observation-simulation pairs.

Supplementary References

1. Yang, H. J. et al. Community structure and composition in response to climate change in a temperate steppe. *Glob. Chang. Biol.* **17**, 452-465 (2011).
2. Kou, D. et al. Dryland soils in northern China sequester carbon during the early 2000s warming hiatus period. *Funct. Ecol.* **32**, 1620-1630 (2018).
3. Zhang, L., Wang, X., Wang, Q. & Jin, L. Advances in the study of arbuscular mycorrhizal fungi in high altitude and cold habitats on Tibetan Plateau. *J. Fungal Res.* **15**, 58-69 (2017).
4. Hedges, L. V., Gurevitch, J. & Curtis, P. S. The meta-analysis of response ratios in experimental ecology. *Ecology* **80**, 1150-1156 (1999).
5. Adams, D. C., Gurevitch, J. & Rosenberg, M. S. Resampling tests for meta-analysis of ecological data. *Ecology* **78**, 1277-1283 (1997).
6. Zhao, L. et al. Permafrost changes and its effects on hydrological processes on Qinghai-Tibet Plateau. *Bulletin of Chinese Academy of Sciences* **34**, 1233-1246 (2019).
7. Saha, S. et al. NCEP Climate Forecast System Version 2 (CFSv2) 6-hourly Products.). Research Data Archive at the National Center for Atmospheric Research, Computational and Information Systems Laboratory (2011).
8. Hastings, M. G., Jarvis, J. C. & Steig, E. J. Anthropogenic impacts on nitrogen isotopes of ice-core nitrate. *Science* **324**, 1288-1288 (2009).
9. Geng, L. et al. Nitrogen isotopes in ice core nitrate linked to anthropogenic atmospheric acidity change. *Proc. Natl Acad. Sci. USA* **111**, 5808 (2014).
10. Vega, C. P. et al. First ice core records of NO_3^- stable isotopes from Lomonosovfonna, Svalbard. *J. Geophys. Res. Atmos.* **120**, 313-330 (2015).
11. Li, Z. et al. Isotopic evidence that recent agriculture overprints climate variability

in nitrogen deposition to the Tibetan Plateau. *Environ. Int.* **138**, 105614 (2020).

12. Crowther, T. W. et al. Quantifying global soil carbon losses in response to warming. *Nature* **540**, 104 (2016).

13. Wilcoxon, F. Individual comparisons by ranking methods. In: *Breakthroughs in Statistics*. (Springer Science & Business Media, 1992).

14. Weil, R. & Brady, N. *The Nature and Properties of Soils. 15th edition*. (Prentice Hall Upper Saddle River, 2017).
