# Supplementary material for: Progressive nitrogen limitation across the Tibetan alpine permafrost region
Source: Nat Commun. 2020 Jul 3;11:3331. doi: 10.1038/s41467-020-17169-6 (PMC7335038; doi:10.1038/s41467-020-17169-6)
Supplement: Supplementary file 2 — Peer Review File [file 41467_2020_17169_MOESM2_ESM.docx]

Peer Review Information

**Journal:** Nature Communications

**Manuscript title:** Progressive nitrogen limitation across the Tibetan alpine permafrost region

**Corresponding author name(s):** Prof Yuanhe Yang

**Editorial notes:**

**Transferred manuscripts**

This manuscript has been previously reviewed at another journal that is not operating a transparent peer review scheme. This document only contains reviewer comments and rebuttal letters for versions considered at *Nature Communications*.

**Reviewer comments & decisions:**

**Reviewer comments, first version:**

Reviewer #1 (Remarks to the Author):

This submission by Kou et al. is extremely provocative and potentially worthy of publication in a Nature series journal. The first sentence of the abstract highlights a key point; namely, that N dynamics need to be considered carefully if we are to understand C dynamics in permafrost settings. The authors’ contention that the research community assumes N availability will increase in degrading permafrost settings is correct. Therefore, their caution that we must fully consider N supply and demand in assessing nutrient limitation is quite compelling.

My main concern with this submission is that the measured signal is very small, with median difference in plant δ15N between decades being only 0.3 ‰. The large number of replicate sites, however, does appear to have yielded a statistically significant result and the modeling provides relatively compelling support that nutrient limitation has increased over time (but see caveats below regarding soil moisture).

Given the small signal, I would advise the authors to make three improvements to the manuscript to better support their argument.

1. Show us the distributions of plant δ15N as they did in Figure 4 for soil. In addition, tell us what percentage of sites saw increases in δ15N versus decreases. More for curiosity sake, if you randomly started removing sites from the data set, at what point would the findings lose statistical significance? This could be useful information for researchers trying to carry out similar studies elsewhere.

2. While the fact that atmospheric N deposition hasn’t changed over time is discussed, is there any possibility that δ15N of such deposition has changed over time, particularly given that sites were sampled along a highway where pollution levels may have changed over time (with decrease in some other atmospheric deposition source offsetting the overall flux)? At the very least, some discussion of the relative stability of δ15N of atmospheric inputs is warranted.

3. The authors mention that there has been significant permafrost degradation in the study region. Permafrost degradation is well known to change soil moisture distribution (independent of precipitation), a key factor in denitrification. Was soil moisture measured or considered in the model, or was precipitation used as a proxy for soil moisture? It is unreasonable to attribute an increase in denitrification and associated gaseous nitrogen loss to increased temperature without considering soil moisture explicitly. This is the most significant weakness in the manuscript as presented.

With these three additions, I would be more fully convinced that the signals observed did indeed represent an increase in N limitation, which would represent a very exciting and profound result.

On a final note, please clarify what soil δ15N represents (i.e. total inorganic and organic N pools in the soil).


Reviewer #2 (Remarks to the Author):

The manuscript by Dan Kou et al. on ‘Progressive nitrogen limitation across the Tibetan alpine permafrost region’ is an interesting and well written paper utilising a large and hard-won dataset. The scope of the paper (a resurveying of 100+ plant survey sites across the Tibeten plateau) is to be commended and the data are interesting with the authors showing an average depletion of plant tissue δ15N over the 10 year time period. The authors explain this observation with a nitrogen limitation hypothesis: that elevated N demand (greater productivity) and N losses to the atmosphere results in progressive reliance on mycorrhizal fungi for N acquisition. Mycorrhizal fungi supply N with more depleted 15N and this is the mechanism by which the authors link their δ15N signal with their hypothesis: greater reliance on mycorrhizal fungi to supplement their increased N demand. The authors combine these data with a biochemical model based on inputs of N2O emissions and various ecosystem properties from the literature and their research sites. I cannot comment properly on the modelling side of this paper as it is quite outside my expertise.

Although the dataset from the repeat surveys is impressive I think the authors overstretch their interpretation of it. Firstly, the authors compare 15N of the plant communities at their sites between the 2000s and the 2010s but provide no detail as to how the plant biomass was selected for δ15N analysis. This is critical because depending on which species from the community is selected for analysis (or which species are pooled), you will get very different δ15N results (Hobbie & Hobbie, 2006). In order to make an estimate of ‘degree of mycorrhizal symbiosis’ the analysis would need to be carried out on a species level and across a number of species. Even then, I find it too speculative as a core principle of the paper to link the hypothesis of N limitation to δ15N via mycorrhizal N supply. In order to link what was measured via mycorrhizal symbiosis, at least one measurement of the symbiosis would need to be conducted. At present there are none. I think it is fine to speculate about the mycorrhizal fungi in the discussion of the results but not as an intermediary mechanism that is taken as certain.

Secondly, if δ15N patterns that the authors measure are in fact driven by an increased reliance on mycorrhizal fungi, this is not necessarily due to N limitation. There are a range of plausible reasons why a plant may invest more into a mycorrhizal symbiosis: phosphorus limitation, water stress, pathogen or toxin defence. I think P limitation would be the most plausible of the above but a number of factors could act in concert to increase mycorrhizal reliance. If the authors had measured N:P ratios in the soil to test whether one of the nutrients had become more limiting over the time course I would be more convinced of their hypothesis.

As it stands, I find the paper interesting but I think the lack of intermediary evidence leads to too many assumptions about the mechanism for the patterns that they observe.

More specific comments on a line by line basis are below:

L74: This last sentence is unclear
L112: ‘lower’ not ‘smaller’
L113: Given that all plants are mycorrhizal, which is not true. Also, plants form mycorrhizas for more reasons. If P limitation is occurring, this would be mistaken for N limitation in their logic
L121: Nice clear hypothesis, well based on literature and important ecosystem processes. I am not convinced the data can address the hypothesis though
L133: a positive d15N would indicate not much mycorrhizal association would it not? Therefore the measurement of a heavily mycorrhizal plant would affect the community average significantly. Community composition shifts over the 10 years need to be taken into account
L302: Was this the same 10x10m plot or slightly different? Were the plots marked with stakes? Even a slight change in plot coverage could change the plant composition and therefore the δ15N
L310:This is an important point but how specific can they be? Where are the vegetation data? Even if the community has not changed, random sampling within the plot could provide shifts isotopes. These criticisms could be headed off with some more specific data to show that the vegetation had not changed.
L314: How accurate is the GPS? Difference of 10 cm could shift the plant community very significantly. Were the sites physically marked?
L323: ‘Ground’ instead of ‘grinded’ how were they ground? Ball mill?
L325: Over what area were these isotope analysese conducted? Was it an average of the 10x10m plot? Which species went into the determination? This is so important
L348 and throughout: ‘Literature’ instead of ‘Literatures’

Literature cited:

Hobbie, J. E., & Hobbie, E. A. (2006). 15N in Symbiotic Fungi and Plants Estimates Nitrogen and Carbon Flux Rates in Arctic Tundra. Ecology, 87(4), 816–822. doi: 10.1890/0012-9658(2006)87[816:NISFAP]2.0.CO;2

**Author rebuttal, first version:**

**Responses to Reviewer #1**

***[Comment 1]*** *This submission by Kou et al. is extremely provocative and potentially worthy of publication in a Nature series journal. The first sentence of the abstract highlights a key point; namely, that N dynamics need to be considered carefully if we are to understand C dynamics in permafrost settings. The authors’ contention that the research community assumes N availability will increase in degrading permafrost settings is correct. Therefore, their caution that we must fully consider N supply and demand in assessing nutrient limitation is quite compelling.* *My main concern with this submission is that the measured signal is very small, with median difference in plant δ15N between decades being only 0.3 ‰. The large number of replicate sites, however, does appear to have yielded a statistically significant result and the modeling provides relatively compelling support that nutrient limitation has increased over time (but see caveats below regarding soil moisture). Given the small signal, I would advise the authors to make three improvements to the manuscript to better support their argument.*

**[Response]** Thanks for the reviewer’s excellent comments. These comments, together with those listed below inspired us to have a deeper thinking on this issue, and thus guided us to conduct a thorough revision of the original MS. By addressing the reviewer’s suggestions listed below, we made the following three major changes:

**First,** we showed more supporting data for the observed changes in plant δ^15^N (*i.e.*, frequency distributions of plant δ^15^N in the 2000s and the 2010s, the proportion of decreased or increased plant δ^15^N among the 107 sampling sites, and the minimum sample size to detect the significant decrease in plant δ^15^N; Figs. R1-2).

**Second,** we added an independent isotopic evidence derived from an ice core across the study area, which demonstrated the relative stability of δ^15^N of atmospheric N deposition (Fig. R3). This additional ice core evidence, together with the relatively stable atmospheric N deposition rate reported in previous studies (Yu et al., 2019b), demonstrates that changes in plant δ^15^N cannot be ascribed to the dynamic atmospheric N deposition during the study period.

**Third,** we provided four independent evidences for soil moisture dynamics on the Tibetan Plateau over the detection period. These four lines of evidence consistently illustrated that soil moisture did not exhibit any significant change over the study period (Figs. R4-5). In addition, we also clearly described how DNDC model considered soil moisture and its potential role in regulating gaseous N loss across our study area.

By doing so, we felt that the revised MS had been significantly improved. Particularly, these additional datasets and analyses provided additional support on our opinion that ‘*N limitation has increased on the Tibetan Plateau during the past decade*’. Thank you! Additionally, we would like to mention that the declining rate in plant δ^15^N (-0.02‰ yr^-1^) calculated based on the median difference (0.3‰) on the Tibetan Plateau is comparable to that reported in other studies, falling in the range between the decreasing rate (-0.01‰ yr^-1^) in temperate forests of the United States (McLauchlan et al., 2017) and the decreasing rate (-0.06‰ yr^-1^) in central North American grasslands (McLauchlan et al., 2010). For the detailed response to each comment, please see below.

***[Comment 2]*** *1. Show us the distributions of plant δ^15^N as they did in Figure 4 for soil. In addition, tell us what percentage of sites saw increases in δ^15^N versus decreases. More for curiosity sake, if you randomly started removing sites from the data set, at what point would the findings lose statistical significance? This could be useful information for researchers trying to carry out similar studies elsewhere.*
**[Response]** Following the reviewer’s suggestions, we provided frequency distributions of plant δ^15^N in the revised MS (Fig. R1), and analyzed the proportion of resampling sites showing increases in plant δ^15^N versus decreases across 107 sampling sites on the Tibetan Plateau. Results showed that plant δ^15^N tended to decrease among 73 resampling sites, accounting for 68% of the total 107 sites. Following the reviewer’s comments, we further used the bootstrap approach (Crowther et al., 2016) to estimate the threshold that made the findings of changes in plant δ^15^N lose statistical significance once we randomly removed sites from the dataset. In our analyses, the bootstrap was iterated for 10,000 times, in each of which the resampling sites were removed randomly and the slope of the linear mixed-effects model used to test changes in plant δ^15^N was estimated based on the remaining sites (Crowther et al., 2016). The estimated slopes were then used to calculate interquartile range for each number of resampling sites that were removed from the dataset (Crowther et al., 2016). The average slope was considered non-significant if the interquartile range of the slope overlapped with the horizontal zero line (Crowther et al., 2016). **Our results showed that the average slope remained significant until >74 resampling sites had been removed from the initial analysis (with 107 resampling sites)** (Fig. R2). These additional analyses demonstrated the robustness of our conclusion about the decreased plant δ^15^N on the Tibetan Plateau over the detection period. We have added these additional analyses in the revised MS (Page 6, lines 112-113, 115-119).

**Fig. R1.** Frequency distributions of plant δ^15^N (nitrogen) in the 2000s and the 2010s derived from the large-scale resampling investigation on the Tibetan Plateau. *N*, the number of sites used for analyzing plant δ^15^N; *Median*, median value of plant δ^15^N.

**Fig. R2.** Bootstrapped estimates of model slope based on different number of resampling sites, of which the model refers to the linear mixed-effects model used to statistically analyze changes in plant δ^15^N (nitrogen) on the Tibetan Plateau over the period from the 2000s to the 2010s. This analysis was used to estimate the threshold that made the findings of changes in plant δ^15^N lose statistical significance once we randomly removed sites from the original dataset. In the analyses, the bootstrap was iterated for 10,000 times, in each of which the resampling sites were removed randomly and the slope of the linear mixed-effects model used to test changes in plant δ^15^N was estimated based on the remaining sites (Crowther et al., 2016). The estimated slopes were then used to calculate interquartile range for each number of resampling sites that were removed from the dataset (Crowther et al., 2016). The average slope was considered non-significant if the interquartile range of the slope overlapped with the horizontal zero line (Crowther et al., 2016). The dashed line represents the first interquartile range which overlapped with the horizontal zero line. Each point in the diagram represents a bootstrapped estimate of model slope, and each box-plot presents the interquartile range and median slope for each number of resampling sites removed from the analysis.

***[Comment 3]*** *2. While the fact that atmospheric N deposition hasn’t changed over time is discussed, is there any possibility that δ15N of such deposition has changed over time, particularly given that sites were sampled along a highway where pollution levels may have changed over time (with decrease in some other atmospheric deposition source offsetting the overall flux)? At the very least, some discussion of the relative stability of δ15N of atmospheric inputs is warranted.*

**[Response]** Following the reviewer’s comment, we tried our best to collect evidences that revealed the temporal dynamics in δ^15^N of atmospheric N deposition on the Tibetan Plateau. Because of the technical barriers and tough conditions, it is hard to find direct evidence for long-term dynamics of δ^15^N-NH_x_ or δ^15^N-NO_x_ derived from precipitation or dry deposition across our study area (this kind of data is also scarce around the world). However, we obtained a recent evidence measured the dynamics of δ^15^N-NH_4_ or δ^15^N-NO_3_ based on an ice core (Li, 2019; Li et al., 2020), which has also been frequently used to reflect the dynamics of atmospheric N deposition and its isotopic characteristics in other regions, such as Greenland and Svalbard (Geng et al., 2014; Hastings et al., 2009; Vega et al., 2015). **The ice core evidence showed that atmospheric δ^15^N did not exhibit significant change since 2000** (Figs. R3a-b). Besides the δ^15^N, the ice core evidence also revealed that neither ammonium nor nitrate concentration experienced significant change during the period from the 2000s to the 2010s (Figs. R3c-d), which confirmed the stabilization of atmospheric N deposition reported by Yu et al. (2019). **Overall, these evidences jointly indicated that both the atmospheric N deposition rate and the corresponding δ^15^N values did not change significantly on the Tibetan Plateau over the detection period.** This finding, together with the small N deposition rate on the Tibetan Plateau (Liu et al., 2015), indicates that atmospheric N deposition is not the main reason for the detected change in plant δ^15^N across our study area. We have provided above-mentioned information in the revised MS (Page 6, lines 107-108; Page 7, lines 123-126; Page 11, lines 225-226; Page 13, line 282).

**Fig. R3.** Changes in δ^15^N-NH_4_^+^ (a), δ^15^N-NO_3_^-^ (b), ammonium (NH_4_^+^) concentration (c) and nitrate (NO_3_^-^) concentration (d) in an ice core on the Tibetan Plateau during the period from 2001 to 2011. The N concentration and δ^15^N in ice cores have widely used to reflect the dynamics of atmospheric nitrogen (N) deposition and its isotopic characteristics (Geng et al., 2014; Hastings et al., 2009; Vega et al., 2015). In our case, to characterize changes in δ^15^N values of atmospheric N deposition, we obtained isotopic observations based on an ice core from our co-author (Prof. Yunting Fang). The changes in N concentration and δ^15^N were examined with Ordinary Least Square regressions, and the dashed fitted lines denote insignificant changes.

***[Comment 4]*** *3. The authors mention that there has been significant permafrost degradation in the study region. Permafrost degradation is well known to change soil moisture distribution (independent of precipitation), a key factor in denitrification. Was soil moisture measured or considered in the model, or was precipitation used as a proxy for soil moisture? It is unreasonable to attribute an increase in denitrification and associated gaseous nitrogen loss to increased temperature without considering soil moisture explicitly. This is the most significant weakness in the manuscript as presented.*

**[Response]** We totally agree with the reviewer that it is oversimplified to attribute the increased gaseous N loss to climate warming without considering soil moisture. The reviewer’s comments could be summarized as the following three issues: (*i*) was soil moisture measured across the study area? (*ii*) was soil moisture considered in the model? (*iii*) what was the potential effect of changes in soil moisture on gaseous N loss over the detection period?

***Issue 1: Was soil moisture measured across the study area?***

Yes, the gravimetric soil moisture content within the 10 cm depth was measured during the two sampling campaigns. By analyzing the observed dataset, we found that the surface soil moisture on the Tibetan Plateau did not exhibit any significant differences between the two sampling periods (Fig. R4). To further confirm this point, we collected three other independent data sources to explore soil moisture dynamics on the Tibetan Plateau over 2000-2015. **The first data source** was purchased from China Meteorological Administration (http://data.cma.cn/), where provided us all the stored soil moisture observations for 7 meteorological stations across the Tibetan Plateau over 2000-2010. Results showed that the average surface (0-10 cm) soil moisture on the Tibetan Plateau did not change over the detection period (Fig. R5a). **The second data source** was derived from a recent study exploring *permafrost changes and its effects on hydrological processes on the Qinghai-Tibetan Plateau*, which provided the observed dataset for soil moisture dynamics in the hinterland of the Tibetan alpine permafrost region (Zhao et al., 2019). By averaging the data from five observation sites, results showed that the surface soil moisture remained stable over the detection period (Fig. R5b). **The third data source** was derived from a global dataset, CFSV2 (NCEP Climate Forecast System Version 2) (Saha et al., 2011). From the CFSV2 dataset, we extracted the surface (5 cm) volumetric soil water content for the 107 resampling sites over 2000-2015, and also found that the surface soil water content did not exhibit any significant change on the Tibetan Plateau (Fig. R5c). **Collectively, results from these four data sources consistently indicated that the surface soil moisture was relatively stable on the Tibetan Plateau from the 2000s to the 2010s.**

**Fig. R4.** Changes in gravimetric soil moisture in the top 10 cm on the Tibetan Plateau during the period from the 2000s to the 2010s. Soil moisture data were derived from the resampling investigations conducted in this study. Soil moisture dynamics was examined with linear mixed-effects model. Numbers near each box diagram denote the percentile of 95%, 75%, 50%, 25% and 5%, respectively.

**Fig. R5.** Changes in soil moisture within the top 10 cm depth derived from China Meteorological Administration (<http://data.cma.cn/>, a), Zhao et al. (2019) (b), CFSV2 (NCEP Climate Forecast System Version 2, <https://rda.ucar.edu/datasets/ds094.0/>, c) and the output of DeNitrification-DeComposition (DNDC) model (d) on the Tibetan Plateau from the 2000s to the 2010s. Based on these datasets, changes in soil moisture were examined with Ordinary Least Square regressions, and the dashed fitted lines in the diagram denoted insignificant trends. GSM: gravimetric soil moisture; VSM, volumetric soil moisture; WFPS, water-filled pore space.

***Issue 2: Was soil moisture considered in the model?***

Yes, soil moisture is simulated by the hydrology submodule in the DNDC model, and is jointly determined by precipitation and other hydrological processes (*e.g.*, runoff, transpiration, evaporation, infiltration and drainage) (Deng et al., 2011; Li et al., 2006; Yu et al., 2019a; Zhang et al., 2002). Numerous previous studies have demonstrated that the DNDC model can well characterize the dynamics of soil moisture in both agricultural and natural ecosystems (Deng et al., 2015; Guest et al., 2018; Li et al., 2019; Li et al., 2017; Tonitto et al., 2010; Yu et al., 2019a). In our case, the data-model comparison also revealed that the model can well capture the observed soil moisture derived from the 107 resampling sites on the Tibetan Plateau (Fig. R6). Besides the spatial variation, we also found that the DNDC model can accurately characterize the temporal dynamics of soil moisture on the Tibetan Plateau, since both the simulated and observed surface soil water-filled pore space experienced no significant changes over the period from the 2000s to the 2010s (Figs. R4-5). BTW, as a model being initially developed for simulating nitrous oxide emissions in agricultural fields, the DNDC model also has a good performance in simulating the effects of soil moisture on denitrification processes (Li, 2000; Li et al., 1992a, b).

**Fig. R6.** Validation of soil water-filled pore space (WFPS) in the top 10 cm simulated by the DeNitrificaiton-DeComposition (DNDC) model. The observed soil WFPS was calculated based on gravimetric soil moisture and bulk density (Weil and Brady, 2017) detected through the field campaign in the 2010s. Four indicators, including *R^2^* (coefficient of determination), *RMSE* (root mean square error), *RMD* (relative mean deviation), and *ME* (model efficiency), were used to evaluate the model performance. *N*, the number of observation-simulation pairs. The dashed line denotes the 1:1 line.

***Issue 3: What was the potential effect of changes in soil moisture on gaseous N loss over the detection period?***

As shown in *Issue 1* and *Issue 2*, the surface soil moisture on the Tibetan Plateau exhibited no significant changes over the detection period (Figs. R4-5), indicating the limited effect of soil moisture dynamics on the increase in gaseous N loss. To further demonstrate this point, we explored the relationship between each year’s total gaseous N loss from denitrification with the corresponding annual average soil water-filled pore space over the detection period (Fig. R7). Results showed that the established relationship was insignificant (Fig. R7), which provided additional evidence for the argument that topsoil moisture dynamics could not be responsible for the increased gaseous N loss across our study area. BTW, the relatively stable topsoil moisture could be associated with the unchanged precipitation across the study area (Supplementary Fig. 2) and the unique permafrost characteristics on the Tibetan Plateau (Zhao et al., 2019), particularly the deep active layer thickness (*ca.* 2.4 m) (Wu and Zhang, 2010) and low ice content (*ca.* 12%) in the permafrost layer (Zhao et al., 2010). We have added associated information about the three issues in the revised MS (Page 7, lines 144-147; Page 10, lines 206-211; Page 13, lines 283-284; Page 20, lines 449-457; Supplementary Text 3). Thanks for your understanding!

**Fig. R7.** Relationship of each year’s total gaseous nitrogen (N) loss from denitrification with the corresponding annual average soil water-filled pore space over the period from 2001 to 2014, which is used to reflect the effect of soil moisture dynamics on gaseous N loss. Both gaseous N loss and soil water-filled pore space are derived from the simulation output of the DeNitrification-DeComposition (DNDC) model, and their relationship is examined with Least Ordinary Square regression. The dashed fitted line denotes an insignificant relationship.

***[Comment 5]*** *With these three additions, I would be more fully convinced that the signals observed did indeed represent an increase in N limitation, which would represent a very exciting and profound result. On a final note, please clarify what soil δ15N represents (i.e. total inorganic and organic N pools in the soil).*

**[Response]** Thanks again for the reviewer’s three insightful suggestions. As mentioned above, we have provided the associated evidences requested by this reviewer. Additionally, following the reviewer’s final suggestion, we have clarified what soil δ^15^N represents (*i.e., total inorganic and organic N pools in the soil*) in the revised MS (Page 5, lines 95-96)*.* By doing so, we feel that our conclusion ‘*the ecosystem on the Tibetan Plateau is experiencing progressive N limitation*’ becomes more convinced. Thank you!

**Responses to Reviewer #2**

***[Comment 1]*** *The manuscript by Dan Kou et al. on ‘Progressive nitrogen limitation across the Tibetan alpine permafrost region’ is an interesting and well written paper utilising a large and hard-won dataset. The scope of the paper (a resurveying of 100+ plant survey sites across the Tibetan plateau) is to be commended and the data are interesting with the authors showing an average depletion of plant tissue δ^15^N over the 10 year time period. The authors explain this observation with a nitrogen limitation hypothesis: that elevated N demand (greater productivity) and N losses to the atmosphere results in progressive reliance on mycorrhizal fungi for N acquisition. Mycorrhizal fungi supply N with more depleted 15N and this is the mechanism by which the authors link their δ15N signal with their hypothesis: greater reliance on mycorrhizal fungi to supplement their increased N demand. The authors combine these data with a biochemical model based on inputs of N2O emissions and various ecosystem properties from the literature and their research sites. I cannot comment properly on the modelling side of this paper as it is quite outside my expertise.*
**[Response]** Thanks for the reviewer’s positive and insightful comments.

***[Comment 2]*** *Although the dataset from the repeat surveys is impressive, I think the authors overstretch their interpretation of it. Firstly, the authors compare 15N of the plant communities at their sites between the 2000s and the 2010s but provide no detail as to how the plant biomass was selected for δ15N analysis. This is critical because depending on which species from the community is selected for analysis (or which species are pooled), you will get very different δ15N results (Hobbie & Hobbie, 2006). In order to make an estimate of ‘degree of mycorrhizal symbiosis’ the analysis would need to be carried out on a species level and across a number of species. Even then, I find it too speculative as a core principle of the paper to link the hypothesis of N limitation to δ15N via mycorrhizal N supply. In order to link what was measured via mycorrhizal symbiosis, at least one measurement of the symbiosis would need to be conducted. At present there are none.* *I think it is fine to speculate about the mycorrhizal fungi in the discussion of the results but not as an intermediary mechanism that is taken as certain.*

**[Response]** To address the reviewer’s comment about the sampling strategy, **we provided details about the collection of plant biomass for the subsequent δ^15^N analysis.** In our study, plant δ^15^N analysis was conducted over 5 m^2^ area (five 1 × 1 m^2^ squares) for each sampling site or each 10 m × 10 m plot (Fig. R8). Specifically, at each sampling site, we first set up five sampling squares with area of 1 × 1 m^2^ at the four corners and centre of a 10 m × 10 m plot (Fig. R8). We then harvested all the aboveground vegetation in each 1 × 1 m^2^ sampling square, which was ground using a ball mill (Mixer Mill MM 400, Retsch, Germany) in the laboratory. After that, plant samples from the five sampling squares were pooled for plant δ^15^N analysis. Therefore, **the plant δ^15^N measured in our study represented the average signal of the vegetation community in five sampling squares with 1 × 1 m^2^ area**. In other words, the isotopic signal represented information of all the plant species within the vegetation community. We have clearly stated this point in the revised MS (Pages 14-15, lines 318-330; Page 16, lines 358-364).

Given that plant δ^15^N analysis was conducted at the community level, shifts in vegetation composition could affect the averaged community δ^15^N. To test this possibility, we detected whether vegetation composition has changed between the two resampling periods by analyzing changes in species richness and Shannon-Wiener index based on the large-scale resampling vegetation investigations. **Results showed that neither of the two indicators exhibited any changes over the detection period** (Fig. R9), indicating the limited effects of vegetation community on the dynamics of plant δ^15^N observed in this study. Due to the potential effects of heavily mycorrhizal plant on the community average δ^15^N, we further analyzed changes in species richness and the relative cover of five families that could be heavily colonized by mycorrhizae on the Tibetan Plateau (*Gramineae*, *Leguminosae*, *Asteraceae*, *Cyperaceae* and *Rosaceae*) (Zhang et al., 2017) and examined changes in the relative cover of the dominant species in the five families (Zhang et al., 2017). **Both the family- and species-level analyses confirmed that the vegetation community composition was unchanged over the period from the 2000s to the 2010s** (Figs. R10-11). These results eliminated the potential confounding effects of vegetation community shifts on plant δ^15^N dynamics observed in this study, suggesting that the community-level plant δ^15^N dynamics could provide insights on *‘the degree of mycorrhizal symbiosis’*. We have added the related analyses about community dynamics in the revised MS (Page 7, lines 123-126; Page 15, lines 330-343; Supplementary Text 1).

**Fig. R8.** The schematic diagram for the resampling campaign on the Tibetan Plateau.

**Fig. R9.** Changes in species richness (a) and Shannon-Wiener index (b) of the vegetation community across the resampling sites on the Tibetan Plateau during the period from the 2000s to the 2010s. Species richness refers to species number per quadrat, and Shannon-Wiener index (*H*) was calculated as: *H* = -∑*P_i_* ln*P_i_*, where *P_i_* is the relative cover of species *i* (Yang et al., 2011). The change in vegetation composition was examined with linear mixed-effects model. Numbers near each box diagram denote the percentile of 95%, 75%, 50%, 25% and 5%, respectively.

**Fig. R10.** Changes in species richness and the relative cover of five families that can be heavily colonized by mycorrhizae on the Tibetan Plateau over the period from the 2000s to the 2010s. The species richness here refers to species number per quadrat for each of the five families, and the relative cover was calculated as the sum of the relative cover of each species in a specific family (Yang et al., 2011). To conduct the family-level analysis, we selected five families that are heavily colonized by mycorrhizae consisted of *Gramineae*, *Cyperaceae*, *Leguminosae*, *Compositae* and *Rosaceae* (Zhang et al., 2017). The changes in species richness and relative cover were examined with linear mixed-effects models. Points in the plot indicate the estimates of model slopes, and error bars denote 95% confidence intervals. The change in species richness or relative cover is considered non-significant if the 95% confidence interval overlaps with the vertical zero line.

**Fig. R11.** Changes in the relative cover of host plant species that can be heavily colonized by mycorrhizae on the Tibetan Plateau over the period from the 2000s to the 2010s. The host plant species *Elymus nutans*, *Carex moorcroftii*, *Oxytropis tibetica*, *Ligularia virgaurea* and *Potentilla nivea* belong to the plant family *Gramineae*, *Cyperaceae*, *Leguminosae*, *Compositae* and *Rosaceae*, respectively (Zhang et al., 2017). The change in species richness and the relative cover was examined with linear mixed-effects models. Points in the plot indicate the estimates of model slopes, and the error bars denote 95% confidence intervals. The change in the relative cover is considered non-significant if the 95% confidence interval overlaps with the vertical zero line.

To further explore the potential shifts in the degree of mycorrhizal symbiosis under changing environment, we communicated with two mycorrhizal experts on the Tibetan Plateau (**Prof. Huyuan Feng** and **Prof. Yongjun Liu** from Lanzhou University, China) and tried to synthesize long-term observations for changes in mycorrhizal symbiosis from the existing studies but failed to find any of them (this kind of long-term dataset is very scarce). Due to this point, together with the reviewer’s suggestion (“*I think it is fine to speculate about the mycorrhizal fungi in the discussion of the results but not as an intermediary mechanism that is taken as certain*”), **we discussed the potential linkage between plant δ^15^N dynamics and vegetation N limitation via mycorrhizal symbiosis in the *Discussion* section of the MS** instead of taking it as a core principle in the *Introduction* section (Pages 6-8, lines 121-154). The discussion is as follows: “*As a widely used indicator to reflect the supply of N relative to plant N demand, a lower plant δ^15^N generally represents an elevated N limitation (Craine et al., 2009; Craine et al., 2018; Elmore et al., 2016; McLauchlan et al., 2007; McLauchlan et al., 2010; McLauchlan et al., 2017). Due to this point, the decreased plant δ^15^N observed in this study suggests enhanced vegetation N limitation across the Tibetan alpine permafrost region over the last decade.*” “*Changes in plant δ^15^N could be associated with multiple reasons, such as shifts in vegetation community, atmospheric N deposition, biological N fixation, plant N uptake through root or mycorrhizae (Craine et al., 2009; Hobbie and Hogberg, 2012; Hobbie and Hobbie, 2006; McLauchlan et al., 2010). However, both vegetation community (Supplementary Figs. 5-7; Supplementary Text 1) and atmospheric N deposition (both rate and δ^15^N, Supplementary Figs. 2-3) were stabilized over the two sampling periods, and thus had limited effects on the plant δ^15^N dynamics observed in this study. Moreover, biological N fixation should not be the main reason for the decrease in plant δ^15^N since plant δ^15^N also significantly decreased among the investigated sites without Leguminosae (p < 0.05; Supplementary Fig. 8). In addition, plant N uptake through roots could elevate rather than diminish plant δ^15^N because bulk soil δ^15^N increased over the detection period (p < 0.01; Fig. 4) and the fractionation of N isotopes was minimal during root N uptake in natural ecosystems (Hobbie and Hobbie, 2008; Hobbie and Hogberg, 2012; Houlton et al., 2007). In contrast to root N uptake, plant N uptake through mycorrhizae can decrease plant δ^15^N given: (i) the transfer of ^15^N-deleted N to plants by mycorrhizae (Craine et al., 2009; Craine et al., 2018; Hobbie and Hogberg, 2012; Hobbie and Hobbie, 2006); (ii) the crucial role of mycorrhizae in supplying N to plants under N-limited conditions (Craine et al., 2018; Hobbie and Hobbie, 2008; Hobbie and Hobbie, 2006; Schimel and Bennett, 2004); (iii) the widespread distribution of plants associated with mycorrhizae (Zhang et al., 2017) across the 107 resampling sites; (iv) the potentially enhanced mycorrhizal colonization (Supplementary Fig. 9; Supplementary Text 2;****Fig. R12****) driven by the two major environmental changes on the Tibetan Plateau (CO_2_ enrichment and climate warming, Supplementary Fig. 2). Nevertheless, besides N limitation, other factors especially phosphorus (P) limitation and water stress could also make plants to invest more to mycorrhizae (Hobbie and Hobbie, 2008; Jayne and Quigley, 2014; Treseder, 2004). However, soil N:P ratio in the top 10 cm across the study area significantly decreased over the detection period (p < 0.01; Supplementary Fig. 10), indicating that N rather than P limitation was more likely to reduce plant δ^15^N via mycorrhizae. Moreover, topsoil moisture across the study area did not exhibit any significant change over the detection period (Supplementary Figs. 11-12; Supplementary Text 3), reflecting the minimal effect of water stress on the plant δ^15^N dynamics. Taken together, of all the factors considered, an enhancement in reliance on mycorrhizae for N acquisition could be the plausible reason for the decreased plant δ^15^N observed in this study.*” Thanks for your understanding!

**Fig. R12.** The weighted response ratios for indicators reflecting experimental warming or CO_2_ enrichment effects on mycorrhizal symbiosis, analyzed based on meta-analyses of published studies (Supplementary text 2; Supplementary Reference Lists 2-3). The *all indicators* in the plot means all the collected variables associated with mycorrhizal symbiosis. *RLC* represents the percentage of root length colonized. *EMH* denotes extraradical mycorrhizal hyphal. Points in the plot denote ln-transformed weighted response ratios, and error bars represent 95% confidence intervals. The vertical dashed line is drawn at the weighted response ratio = 0. The warming or CO_2_ enrichment effect on mycorrhizal symbiosis was considered non-significant if the 95% confidence interval overlapped with the dashed vertical line.

***[Comment 3]*** *Secondly, if δ15N patterns that the authors measure are in fact driven by an increased reliance on mycorrhizal fungi, this is not necessarily due to N limitation. There are a range of plausible reasons why a plant may invest more into a mycorrhizal symbiosis: phosphorus limitation, water stress, pathogen or toxin defence. I think P limitation would be the most plausible of the above but a number of factors could act in concert to increase mycorrhizal reliance. If the authors had measured N:P ratios in the soil to test whether one of the nutrients had become more limiting over the time course I would be more convinced of their hypothesis. As it stands, I find the paper interesting but I think the lack of intermediary evidence leads to too many assumptions about the mechanism for the patterns that they observe.*

**[Response]** We totally agree with the reviewer that, besides N limitation, other factors especially P limitation could make a plant to invest more into a mycorrhizal symbiosis (Hobbie and Hogberg, 2012). **To test this possibility, we followed the reviewer’s suggestion to measure soil N:P ratios with the samples derived from the repeated surveys.** Based on these additional measurements, we then tested whether one of the nutrients on the Tibetan Plateau had become more limiting over the detection period. Our results showed that the mean N:P ratio in the surface (0-10 cm) soil was 4.4 in the 2010s, which was lower than the mean value 5.3 in the 2000s (Fig. R13). **Further analysis revealed that the decrease in soil N:P ratio on the Tibetan Plateau was significant (*p* < 0.01, Fig. R13) over the period from the 2000s to the 2010s.** These results highlighted that N rather than P had become more limiting over the time course, which provide another support for our opinion that progressive N limitation was occurring on the Tibetan Plateau. Besides P limitation, we also explored the effects of water stress on mycorrhizal symbiosis over the detection period. Nevertheless, given that soil moisture did not exhibit significant change over the detection period (Figs. R4-5), changes in soil water content may not be a plausible reason for an increased mycorrhizal dependence. Additionally, we would like to mention that the observations for the dynamics of pathogen and toxin defence and their effects on mycorrhizal symbiosis are scarce across the study area, which needs to be addressed in future studies for better understanding their potential role in regulating mycorrhizal dynamics. We have added the information concerning the effects of P limitation and water stress in the revised MS (Pages 7-8, lines 139-147). Thanks for your understanding!

Following the comments from this reviewer and *Reviewer#1*, **we have added multiple intermediary evidences in the revised MS for the potential mechanisms about the decreased plant δ^15^N pattern observed in our study**, such as changes in soil N:P ratio, changes in soil moisture, changes in vegetation community, potential effects of CO_2_ enrichment and warming on mycorrhizal colonization and changes in indicators representing δ^15^N of atmospheric N deposition. Based on these intermediary evidences, **we discussed the potential linkage between plant δ^15^N dynamics and vegetation N limitation via mycorrhizal symbiosis in the *Discussion* section of the MS** (Pages 6-8, lines 121-149) as follows: “*Changes in plant δ^15^N could be associated with multiple reasons, such as shifts in vegetation community, atmospheric N deposition, biological N fixation, plant N uptake through root or mycorrhizae (Craine et al., 2009; Hobbie and Hogberg, 2012; Hobbie and Hobbie, 2006; McLauchlan et al., 2010). However, both vegetation community (Supplementary Figs. 5-7; Supplementary Text 1) and atmospheric N deposition (both rate and δ^15^N, Supplementary Figs. 2-3) were stabilized over the two sampling periods, and thus had limited effects on the plant δ^15^N dynamics observed in this study. Moreover, biological N fixation should not be the main reason for the decrease in plant δ^15^N since plant δ^15^N also significantly decreased among the investigated sites without Leguminosae (p < 0.05; Supplementary Fig. 8). In addition, plant N uptake through roots could elevate rather than diminish plant δ^15^N because bulk soil δ^15^N increased over the detection period (p < 0.01; Fig. 4) and the fractionation of N isotopes was minimal during root N uptake in natural ecosystems (Hobbie and Hobbie, 2008; Hobbie and Hogberg, 2012; Houlton et al., 2007). In contrast to root N uptake, plant N uptake through mycorrhizae can decrease plant δ^15^N given: (i) the transfer of ^15^N-deleted N to plants by mycorrhizae (Craine et al., 2009; Craine et al., 2018; Hobbie and Hogberg, 2012; Hobbie and Hobbie, 2006); (ii) the crucial role of mycorrhizae in supplying N to plants under N-limited conditions (Craine et al., 2018; Hobbie and Hobbie, 2008; Hobbie and Hobbie, 2006; Schimel and Bennett, 2004); (iii) the widespread distribution of plants associated with mycorrhizae (Zhang et al., 2017) across the 107 resampling sites; (iv) the potentially enhanced mycorrhizal colonization (Supplementary Fig. 9; Supplementary Text 2) driven by the two major environmental changes on the Tibetan Plateau (CO_2_ enrichment and climate warming, Supplementary Fig. 2). Nevertheless, besides N limitation, other factors especially phosphorus (P) limitation and water stress could also make plants to invest more to mycorrhizae (Hobbie and Hobbie, 2008; Jayne and Quigley, 2014; Treseder, 2004). However, soil N:P ratio in the top 10 cm across the study area significantly decreased over the detection period (p < 0.01; Supplementary Fig. 10), indicating that N rather than P limitation was more likely to reduce plant δ^15^N via mycorrhizae. Moreover, topsoil moisture across the study area did not exhibit any significant change over the detection period (Supplementary Figs. 11-12; Supplementary Text 3), reflecting the minimal effect of water stress on the plant δ^15^N dynamics. Taken together, of all the factors considered, an enhancement in reliance on mycorrhizae for N acquisition could be the plausible reason for the decreased plant δ^15^N observed in this study.”*

Last but not the least, we would like to mention that the decreased plant δ^15^N has been widely used to indicate the reduced N availability in previous studies (Craine et al., 2018; Hobbie and Hogberg, 2012; McLauchlan et al., 2010). Moreover, **we would also like to mention that plant δ^15^N was just one of the multiple evidences showing the progressive N limitation occurred on the Tibetan Plateau. Besides it, our opinion was also supported by other evidences,** including the decrease in soil N:P ratio, the increased plant N stress index, the increase in plant N demand, the increase in gaseous N loss and the increase in soil δ^15^N. Thanks for your understanding!

**Fig. R13.** Decadal change in the ratio of soil nitrogen (N) content to phosphorus (P) content (N:P ratio) in the top 10 cm on the Tibetan Plateau over the period from the 2000s to the 2010s. Soil N and P contents were measured based on samples collected across the 107 resampling sites. The change in soil N:P ratio was examined with Wilcoxon signed rank test due to the skewed distributions (Wilcoxon, 1992). Points in the interval plot denote mean values and error bars represent 95% confidence intervals.

***[Comment 4]*** *L74: This last sentence is unclear.*

**[Response]** Sorry for the confusion. We have clarified this sentence by rephrasing it as follows: “*Therefore, deeper exploration of ecosystem N dynamics in permafrost regions is imperative to better predict permafrost C cycle and its feedback to climate warming*” (Page 4, lines 67-69).

***[Comment 5]*** *L112: ‘lower’ not ‘smaller’.*

**[Response]** Done as suggested (Page 8, lines 151-152).

***[Comment 6]*** *L113: Given that all plants are mycorrhizal, which is not true. Also, plants form mycorrhizas for more reasons. If P limitation is occurring, this would be mistaken for N limitation in their logic.*

**[Response]** Sorry for this mistake. Following the reviewer’s current comment and also previous one (‘*I think it is fine to speculate about the mycorrhizal fungi in the discussion of the results but not as an intermediary mechanism that is taken as certain.’*), **we deleted the original hypothesis that assumed all plants are mycorrhizal in the revised MS**. Additionally, we agree with the reviewer that plants form mycorrhizas for more reasons, including P limitation et al. As mentioned above, to test whether P limitation becomes more limiting over the detection period, we examined changes in soil N:P ratio on the Tibetan Plateau over the period from the 2000s to the 2010s. **The decreased soil N:P ratio (*p* < 0.01, Fig. R13) demonstrated that N rather than P became more limited over the time course, suggesting that the effect of P limitation on mycorrhizal symbiosis is relatively weak across our study area**. This additional evidence, together with other evidences including the decrease in plant δ^15^N, the increase in plant N stress revealed by the modelling analysis, jointly demonstrated the progressive N limitation occurred on the Tibetan Plateau. We have added the discussions concerning the potential effects of P limitation on mycorrhizal symbiosis in the revised MS (Page 7, lines 139-144).

***[Comment 7]*** L121: *Nice clear hypothesis, well based on literature and important ecosystem processes. I am not convinced the data can address the hypothesis though.*

**[Response]** As mentioned above, following the reviewer’s current comment and also previous one (‘*I think it is fine to speculate about the mycorrhizal fungi in the discussion of the results but not as an intermediary mechanism that is taken as certain.’*), we have removed the original hypothesis from the *Introduction* section, and discussed the potential reasons for the observed plant δ^15^N dynamics in the *Discussion* section of the revised MS (Pages 6-8, lines 121-154).

***[Comment 8]*** *L133: a positive d15N would indicate not much mycorrhizal association would it not? Therefore, the measurement of a heavily mycorrhizal plant would affect the community average significantly. Community composition shifts over the 10 years need to be taken into account.*

**[Response]** Yes, we agree with the reviewer that a higher plant δ^15^N would indicate less mycorrhizal association (Hobbie and Hobbie, 2006) and the measurement of a heavily mycorrhizal plant would play a significant role in affecting the community average. Due to this point, we not only detected the changes in vegetation community composition on the community level, but also on the family and species level that are heavily infected by mycorrhiza (Zhang et al., 2017). **Our additional analyses showed that vegetation community had not shifted at any of the three levels over the detection period** (Figs. R9-11). We have added the associated information in the revised MS (Page 7, lines 123-126; Page 15, lines 330-343; Supplementary Text 1).

***[Comment 9]*** *L302: Was this the same 10x10m plot or slightly different? Were the plots marked with stakes? Even a slight change in plot coverage could change the plant composition and therefore the δ15N.*

**[Response]** As mentioned above, the plot was marked with the original soil pit (Fig. R8). With the help of GPS and the original soil pit as well as the people participated in the original sampling, **the same 10 m × 10 m plot was investigated** during the resampling campaigns (Ding et al., 2017). More importantly, as mentioned above, results derived from the resampling campaigns revealed that **vegetation community had not changed at any of the three levels: the community level, family level and species level** (Figs. R9-11). We have provided detailed information concerning the locating of plots during the two sampling periods (Page 14, lines 305-312) and the analysis of changes in vegetation composition (Page 7, lines 123-126; Page 15, lines 330-343; Supplementary Text 1) in the revised MS.

***[Comment 10]*** *L310: This is an important point but how specific can they be? Where are the vegetation data? Even if the community has not changed, random sampling within the plot could provide shifts isotopes. These criticisms could be headed off with some more specific data to show that the vegetation had not changed.*

**[Response]** We agree with the reviewer that the information about changes in vegetation community is important for understanding plant ^15^N patterns observed in this study. As mentioned above, we explored changes in vegetation composition from the following three levels: the community level, family level and species level. **Our additional analyses showed that vegetation community did not exhibit any significant change at any of the three levels** (Figs. R9-11). We have provided the detailed information about vegetation community dynamics in the revised MS (Page 7, lines 123-126; Page 15, lines 330-343; Supplementary Text 1).

We also agree with the reviewer that random sampling within the plot could induce shifts in isotopes even if the community composition has not changed. Nevertheless, we would like to mention that the plant δ^15^N that we measured in this study represented the signal at the community level rather than at the species level (Fig. R8). Therefore, the issue related to the random sampling within plot did not exist in our study. We have added the detailed information about how to collect samples for plant δ^15^N analysis in the revised MS (Pages 14-15, lines 318-330; Page 16, lines 358-364). Thanks for your understanding!

***[Comment 11]*** *L314: How accurate is the GPS? Difference of 10 cm could shift the plant community very significantly. Were the sites physically marked?*

**[Response]** The GPS could locate a site with a decimeter precision (Ding et al., 2017), and the sampling sites were naturally marked by the original soil pit (Fig. R8). During our resampling campaign, we first preliminarily located a resampling site with our GPS. Then, the resampling site was accurately located (the original 10 m × 10 m plot was recovered) by the significant mark of three soil pits excavated during the 2000s (Fig. R8). More importantly, as mentioned above, our results showed that the plant community composition kept stable during our detection period (Figs. R9-11). We have provided the detailed information concerning the locating of plots in the two sampling periods (Page 14, lines 305-312) and the analysis of changes in vegetation composition (Page 7, lines 123-126; Page 15, lines 330-343; Supplementary Text 1) in the revised MS.

***[Comment 12]*** *L323: ‘Ground’ instead of ‘grinded’ how were they ground? Ball mill?*

**[Response]** Done as suggested. Yes, both plant and soil samples were ground with a ball mill (Mixer Mill MM 400, Retsch, Germany) in the laboratory. We have added this information in the revised MS as follows: “*In our study, site-level plant δ^15^N measurement was conducted based on the five vegetation samples collected over the five quadrats (1 m × 1 m) within a sampling plot (10 m × 10 m; Supplementary Fig. 1). Specifically, at each sampling site, plant samples were harvested from each of the five 1 × 1 m^2^ quadrats, dried to a constant weight at 65 °C, and weighed as aboveground biomass separately. The dried plant samples were then* ***ground using a ball mill (Mixer Mill MM 400, Retsch, Germany)*** *for plant δ^15^N analysis. Additionally, the collected soil samples were air dried, passed through a 2 mm sieve (removing gravel and coarse roots), processed to remove fine roots and* ***ground with a ball mill (Mixer Mill MM 400, Retsch, Germany)****.*” (Page 16, lines 358-366).

***[Comment 13]*** *L325: Over what area were these isotope analyses conducted? Was it an average of the 10x10m plot? Which species went into the determination? This is so important.*

**[Response]** As mentioned above, in our study, **plant δ^15^N analysis was conducted over 5 m^2^ area (five 1 × 1 m^2^ squares)** for each sampling site or each 10 m × 10 m plot (Fig. R8). Specifically, at each sampling site, we first set up five sampling squares with area of 1 × 1 m^2^ at the four corners and centre of a 10 m × 10 m plot (Fig. R8). We then harvested all the aboveground vegetation in each 1 × 1 m^2^ sampling square, which was ground using a ball mill (Mixer Mill MM 400, Retsch, Germany) in the laboratory. After that, the plant samples from the five sampling squares were homogenized for plant δ^15^N analysis. Therefore, **the plant δ^15^N measured in our study reflected the average signal of the vegetation community in five sampling squares with 1 × 1 m^2^ area**. In other words, **the isotopic signal represented information of all the plant species within the vegetation community**. We have clearly stated this point in the revised MS (Pages 14-15, lines 318-330; Page 16, lines 358-364).

***[Comment 14]*** *L348 and throughout: ‘Literature’ instead of ‘Literatures’*
**[Response]** Done as suggested (Pages 17-18, lines 388, 393 and 399).

***[Comment 15]*** Literature cited: Hobbie, J. E., & Hobbie, E. A. (2006). 15N in Symbiotic Fungi and Plants Estimates Nitrogen and Carbon Flux Rates in Arctic Tundra. Ecology, 87(4), 816–822. doi: 10.1890/0012-9658(2006)87[816:NISFAP]2.0.CO;2

**[Response]** We have carefully read this paper and cited it in the revised MS.

Thanks again for this reviewer’s insightful and professional review. These comments inspired us to have a deeper thinking on both field sampling and results integration, and thus guided us to conduct a thorough revision of the original MS. To address these insightful comments, **we described the field sampling strategies in more detail, and discussed the potential mechanisms for the observed plant δ^15^N dynamics. Particularly, we added another 13 supplementary figures and three supplementary texts to support the main conclusion drawn in this study**. By doing so, we feel that our revised manuscript has been greatly improved and expect that the reviewer will be satisfied with the revised manuscript. Thank you!

**References**

Craine, J.M., Elmore, A.J., Aidar, M.P.M., et al., 2009. Global patterns of foliar nitrogen isotopes and their relationships with climate, mycorrhizal fungi, foliar nutrient concentrations, and nitrogen availability. New Phytol. 183, 980-992.

Craine, J.M., Elmore, A.J., Wang, L., et al., 2018. Isotopic evidence for oligotrophication of terrestrial ecosystems. Nat. Ecol. Evol. 2, 1735-1744.

Crowther, T.W., Todd-Brown, K.E.O., Rowe, C.W., et al., 2016. Quantifying global soil carbon losses in response to warming. Nature 540, 104.

Deng, J., Li, C., Frolking, S., 2015. Modeling impacts of changes in temperature and water table on C gas fluxes in an Alaskan peatland. J. Geophys. Res. Biogeo. 120, 1279-1295.

Deng, J., Zhu, B., Zhou, Z., et al., 2011. Modeling nitrogen loadings from agricultural soils in southwest China with modified DNDC. J. Geophys. Res. Biogeo. 116, G02020.

Ding, J., Chen, L., Ji, C., et al., 2017. Decadal soil carbon accumulation across Tibetan permafrost regions. Nat. Geosci. 10, 420-424.

Elmore, A.J., Nelson, D.M., Craine, J.M., 2016. Earlier springs are causing reduced nitrogen availability in North American eastern deciduous forests. Nat. Plants 2, 16133.

Geng, L., Alexander, B., Cole-Dai, J., et al., 2014. Nitrogen isotopes in ice core nitrate linked to anthropogenic atmospheric acidity change. Proc. Natl Acad. Sci. USA 111, 5808.

Guest, G., Smith, W., Grant, B., et al., 2018. Comparing the performance of the DNDC, Holos, and VSMB models for predicting the water partitioning of various crops and sites across Canada. Can. J. Soil Sci. 98, 212-231.

Hastings, M.G., Jarvis, J.C., Steig, E.J., 2009. Anthropogenic impacts on nitrogen isotopes of ice-core nitrate. Science 324, 1288-1288.

Hobbie, E.A., Hobbie, J.E., 2008. Natural abundance of N-15 in nitrogen-limited forests and tundra can estimate nitrogen cycling through mycorrhizal fungi: A review. Ecosystems 11, 815-830.

Hobbie, E.A., Hogberg, P., 2012. Nitrogen isotopes link mycorrhizal fungi and plants to nitrogen dynamics. New Phytol. 196, 367-382.

Hobbie, J.E., Hobbie, E.A., 2006. ^15^N in symbiotic fungi and plants estimates nitrogen and carbon flux rates in Arctic tundra. Ecology 87, 816-822.

Houlton, B.Z., Sigman, D.M., Schuur, E.A.G., et al., 2007. A climate-driven switch in plant nitrogen acquisition within tropical forest communities. Proc. Natl Acad. Sci. USA 104, 8902-8906.

Jayne, B., Quigley, M., 2014. Influence of arbuscular mycorrhiza on growth and reproductive response of plants under water deficit: a meta-analysis. Mycorrhiza 24, 109-119.

Li, C., Farahbakhshazad, N., Jaynes, D.B., et al., 2006. Modeling nitrate leaching with a biogeochemical model modified based on observations in a row-crop field in Iowa. Ecol. Modell. 196, 116-130.

Li, C.S., 2000. Modeling trace gas emissions from agricultural ecosystems. Nutr. Cycl. Agroecosystems 58, 259-276.

Li, C.S., Frolking, S., Frolking, T.A., 1992a. A model of nitrous oxide evolution from soil driven by rainfall events: 1. Model structure and sensitivity. J. Geophys. Res. Atmos. 97, 9759-9776.

Li, C.S., Frolking, S., Frolking, T.A., 1992b. A model of nitrous oxide evolution from soil driven by rainfall events: 2. Model applications. J. Geophys. Res. Atmos. 97, 9777-9783.

Li, S., Zheng, X., Zhang, W., et al., 2019. Modeling ammonia volatilization following the application of synthetic fertilizers to cultivated uplands with calcareous soils using an improved DNDC biogeochemistry model. Sci. Total Environ. 660, 931-946.

Li, Z., 2019. Historical nitrogen deposition under conditions of human activity. University of Chinese Academy of Sciences, Beijing.

Li, Z., Hastings, M.G., Walters, W.W., et al., 2020. Isotopic evidence that recent agriculture overprints climate variability in nitrogen deposition to the Tibetan Plateau. Environ. Int. 138, 105614.

Li, Z., Yang, J.Y., Drury, C.F., et al., 2017. Evaluation of the DNDC model for simulating soil temperature, moisture and respiration from monoculture and rotational corn, soybean and winter wheat in Canada. Ecol. Modell. 360, 230-243.

Liu, Y., Xu-Ri, Wang, Y., et al., 2015. Wet deposition of atmospheric inorganic nitrogen at five remote sites in the Tibetan Plateau. Atmospheric Chem. Phys. 15, 11683-11700.

McLauchlan, K.K., Craine, J.M., Oswald, W.W., et al., 2007. Changes in nitrogen cycling during the past century in a northern hardwood forest. Proc. Natl Acad. Sci. USA 104, 7466-7470.

McLauchlan, K.K., Ferguson, C.J., Wilson, I.E., et al., 2010. Thirteen decades of foliar isotopes indicate declining nitrogen availability in central North American grasslands. New Phytol. 187, 1135-1145.

McLauchlan, K.K., Gerhart, L.M., Battles, J.J., et al., 2017. Centennial-scale reductions in nitrogen availability in temperate forests of the United States. Sci. Rep. 7, 7856.

Saha, S., Moorthi, S., Wu, X., et al., 2011. NCEP Climate Forecast System Version 2 (CFSv2) 6-hourly Products. Research Data Archive at the National Center for Atmospheric Research, Computational and Information Systems Laboratory, Boulder, CO.

Schimel, J.P., Bennett, J., 2004. Nitrogen mineralization: Challenges of a changing paradigm. Ecology 85, 591-602.

Tonitto, C., Li, C., Seidel, R., et al., 2010. Application of the DNDC model to the Rodale Institute Farming Systems Trial: challenges for the validation of drainage and nitrate leaching in agroecosystem models. Nutr. Cycl. Agroecosystems 87, 483-494.

Treseder, K.K., 2004. A meta-analysis of mycorrhizal responses to nitrogen, phosphorus, and atmospheric CO_2_ in field studies. New Phytol. 164, 347-355.

Vega, C.P., Pohjola, V.A., Samyn, D., et al., 2015. First ice core records of NO_3_^-^ stable isotopes from Lomonosovfonna, Svalbard. J. Geophys. Res. Atmos. 120, 313-330.

Weil, R., Brady, N., 2017. The Nature and Properties of Soils. 15th edition. Prentice Hall Upper Saddle River, NJ, USA.

Wilcoxon, F., 1992. Individual comparisons by ranking methods, Breakthroughs in Statistics. Springer Science & Business Media, New York, USA, pp. 196-202.

Wu, Q.B., Zhang, T.J., 2010. Changes in active layer thickness over the Qinghai-Tibetan Plateau from 1995 to 2007. J. Geophys. Res. Atmos. 115, D09107.

Yang, H.J., Wu, M.Y., Liu, W.X., et al., 2011. Community structure and composition in response to climate change in a temperate steppe. Glob. Chang. Biol. 17, 452-465.

Yu, C., Huang, X., Chen, H., et al., 2019a. Managing nitrogen to restore water quality in China. Nature 567, 516-520.

Yu, G., Jia, Y., He, N., et al., 2019b. Stabilization of atmospheric nitrogen deposition in China over the past decade. Nat. Geosci. 12, 424-429.

Zhang, L., Wang, X., Wang, Q., et al., 2017. Advances in the study of arbuscular mycorrhizal fungi in high altitude and cold habitats on Tibetan Plateau. J. Fungal Res. 15, 58-69.

Zhang, Y., Li, C.S., Trettin, C.C., et al., 2002. An integrated model of soil, hydrology, and vegetation for carbon dynamics in wetland ecosystems. Global Biogeochem. Cycles 16, 1061.

Zhao, L., Ding, Y., Liu, G., et al., 2010. Estimates of the reserves of ground ice in permafrost regions on the Tibetan Plateau. Journal of Glaciology and Geocryology 32, 1-9.

Zhao, L., Hu, G., Zou, D., et al., 2019. Permafrost changes and its effects on hydrological processes on Qinghai-Tibet Plateau. Bulletin of Chinese Academy of Sciences 34, 1233-1246.

**Reviewer comments, second version:**

Reviewer #1 (Remarks to the Author):

In my previous review of the manuscript "Progressive nitrogen limitation across the Tibetan alpine permafrost region" I indicated that I felt the manuscript was both compelling and provocative, and that it represents an important advance in our understanding of C and N cycling in permafrost landscapes. My main concern was that the authors had not fully eliminated all other possible factors responsible for a relatively small signal and suggested that they consider variability in soil moisture, d15N of atmospheric sources, etc. I am delighted to say that the response to my suggestions was the most thorough I have seen in over 20 years of reviewing manuscripts for publication. I believe all of my concerns have been dealt with admirably.

I was also asked by the editor to consider the authors' response to comments by the second reviewer. While I am clearly less well versed in matters of mycorrhizal associations than Reviewer 2, I felt that that the authors showed the same diligence in assessing questions of changes in plant community composition, N versus P limitation, etc.

As such, I feel this manuscript is now suitable for publication in Nature Communications and will represent a valuable addition to a growing literature on crucial couplings between C and N cycles in permafrost environments.

Jeffrey Heikoop

**Author rebuttal, second version:**

**Response to Reviewer #1**

***[Comment]*** *In my previous review of the manuscript "Progressive nitrogen limitation across the Tibetan alpine permafrost region" I indicated that I felt the manuscript was both compelling and provocative, and that it represents an important advance in our understanding of C and N cycling in permafrost landscapes. My main concern was that the authors had not fully eliminated all other possible factors responsible for a relatively small signal and suggested that they consider variability in soil moisture, d15N of atmospheric sources, etc. I am delighted to say that the response to my suggestions was the most thorough I have seen in over 20 years of reviewing manuscripts for publication. I believe all of my concerns have been dealt with admirably. I was also asked by the editor to consider the authors' response to comments by the second reviewer. While I am clearly less well versed in matters of mycorrhizal associations than Reviewer 2, I felt that that the authors showed the same diligence in assessing questions of changes in plant community composition, N versus P limitation, etc. As such, I feel this manuscript is now suitable for publication in Nature Communications and will represent a valuable addition to a growing literature on crucial couplings between C and N cycles in permafrost environments.*

**[Response]** Thanks for the reviewer’s recognition for our revision.

**Reviewer comments, third version:**

**Author rebuttal, third version:**
